# Geospallins A–C: New Thiodiketopiperazines with Inhibitory Activity against Angiotensin-Converting Enzyme from a Deep-Sea-Derived Fungus *Geosmithia pallida* FS140

**DOI:** 10.3390/md16120464

**Published:** 2018-11-23

**Authors:** Zhang-Hua Sun, Jiangyong Gu, Wei Ye, Liang-Xi Wen, Qi-Bin Lin, Sai-Ni Li, Yu-Chan Chen, Hao-Hua Li, Wei-Min Zhang

**Affiliations:** 1 State Key Laboratory of Applied Microbiology Southern China, Guangdong Provincial Key Laboratory of Microbial Culture Collection and Application, Guangdong Open Laboratory of Applied Microbiology, Guangdong Institute of Microbiology, Guangzhou 510070, China; sysuszh@126.com (Z.-H.S.); yewei@gdim.cn (W.Y.); lisn@gdim.cn (S.-N.L.); yuchan2006@126.com (Y.-C.C.); hhli100@126.com (H.-H.L.); 2 Guangdong Provincial Key Laboratory of New Drug, Development and Research of Chinese Medicine, Mathematical Engineering Academy of Chinese Medicine, Guangzhou University of Chinese Medicine, Guangzhou 510006, China; 3 The Second Institute of Clinical Medicine, Guangzhou University of Chinese Medicine, Guangzhou 510006, China; gujy@gzucm.edu.cn; 4 School of Pharmaceutical Sciences, Guangzhou University of Chinese Medicine, Guangzhou 510006, China; wen_en_liangxi@163.com (L.-X.W.); 15622154060@163.com (Q.-B.L.)

**Keywords:** thiodiketopiperazines, *Geosmithia pallida*, deep-sea-derived fungus

## Abstract

Three new thiodiketopiperazines, geospallins A–C (**1**–**3**), together with nine known analogues (**4**–**12**), were isolated from the culture of the deep-sea sediment-derived fungus *Geosmithia pallida* FS140. Among them, geospallins A and B (**1** and **2**) represent rare examples of thiodiketopiperazines featuring an S-methyl group at C-10 and a tertiary hydroxyl group at C-11. Their structures were determined by high-resolution electrospray mass spectrometry (HRESIMS), spectroscopic analyses, and electronic circular dichroism (ECD) calculations. Their angiotensin-converting enzyme (ACE) inhibitory activity was reported, and geospallins A–C (**1**–**3**) showed inhibitory activity with IC_50_ values of 29–35 µM.

## 1. Introduction

2,5-Diketopiperazines are cyclodipeptides obtained by the condensation of two α-amino acids. This subunit is often found alone or embedded in larger, more complex architectures in a variety of natural products from fungi, bacteria, the plant kingdom, and mammals [1]. They are not only a class of naturally occurring privileged structures that have the ability to bind to a wide range of receptors, but they also have several characteristics that make them attractive scaffolds for drug discovery [2,3]. Thiodiketopiperazines, such as gliotoxins, chetoseminudins, luteoalbusins, chetracins, and apoaranotins, are common across the microbial world and have highly diverse organic structures characterized by sulfur-containing functional groups. They also exhibit various pharmacological activities, such as antifungal, antibacterial, and cytotoxic activities [4]. The angiotensin-converting enzyme (ACE) is an important target and has broad effects in different systems, and ACE inhibitors were originally developed for the treatment of congestive heart failure, diabetic kidney disease, and hypertension management [5]. ACE cleaves many peptides besides angiotensin I and thereby affects diverse physiological functions, including renal development and male reproduction. In addition, ACE has a role in both innate and adaptive responses by modulating macrophage and neutrophil function—effects that are magnified when these cells overexpress ACE. Macrophages that overexpress ACE are more effective against tumors and infections [5]. Sulfur-containing metabolites are crucial for the inhibitory activity against ACE, which catalyzes the reaction from angiotensin I to angiotensin II in the renin–angiotensin system and plays a major role in hypertension [6].

Recently, we initiated the investigation of microorganisms derived from deep-sea sediments, aiming at discovering new metabolites with potent bioactivity [7,8,9]. As part of the program, the fungus *Geosmithia pallida* FS140 was isolated from a sediment collected at a depth of 2403 m in the South China Sea (19°28.581′ N, 115°27.251′ E). Chemical investigation of the fermentation broth led to the isolation of a series of diketopiperazines including three new thiodiketopiperazines, named geospallins A–C (**1**–**3**), as well as nine known analogues (**4**–**12**). Geospallins A and B (**1** and **2**) represent rare examples of thiodiketopiperazines featuring an S-methyl group at C-10 and a tertiary hydroxyl group at C-11. Biological tests verified that compounds **1**−**3** were responsible for the inhibition of ACE. Details of the isolation, structure elucidation, and biological activities of diketopiperazines **1**–**12** are presented below.

## 2. Results

**Figure 1:**
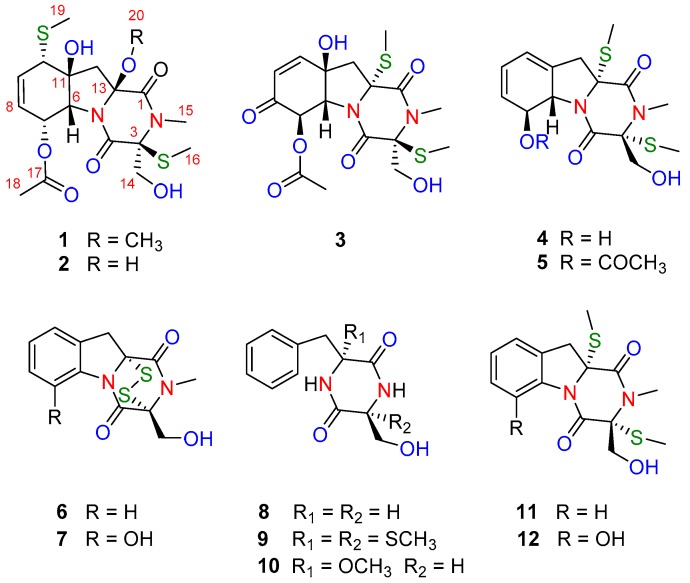
Structures of compounds **1**–**12** isolated from *G. pallida* FS140.

The fermentation broth of the deep-sea-derived fungus *G. pallida* FS140 was extracted with EtOAc and then concentrated under reduced pressure to give an extract. The EtOAc extract was subjected to a series of solvent/solvent partitioning steps to afford compounds **1**–**12** (Figure 1). Three new structures were identified by the combination of spectroscopic analysis, high-resolution electrospray mass spectrometry (HRESIMS), and electronic circular dichroism (ECD) calculation, while twelve known analogues were identified as bisdethiobis (methylthio)gliotoxin (**4**) [10,11], 6-acetylbis(methylthio)gliotoxin (**5**) [9,12], 6-deoxy-5a,6-didehydrogliotoxin (**6**) [13], 5a,6-didehydrogliotoxin (**7**) [14], 6-(phenylmethyl)-(3*R*,6*R*)-2,5-piperazinedione (**8**) [15], 3-(hydroxymethyl)-3,6-bis(methylthio)-6-(phenylmethyl)-(3*R*,6*R*)-2,5-piperazinedione (**9**) [15], 3-(hydroxymethyl)-6-(methoxyl)-6-(phenylmethyl)-(3*R*,6*R*)-2,5-piperazinedione (**10**) [16], 5a,6-anhydrobisdethiobis(methylthio)gliotoxin (**11**) [17], and bisdethiobis (methylthio)gliotoxin (**12**) [12] by comparison of their spectroscopic data with those in the literature. 

### 2.1. Identification of New Compounds

**Figure 2:**
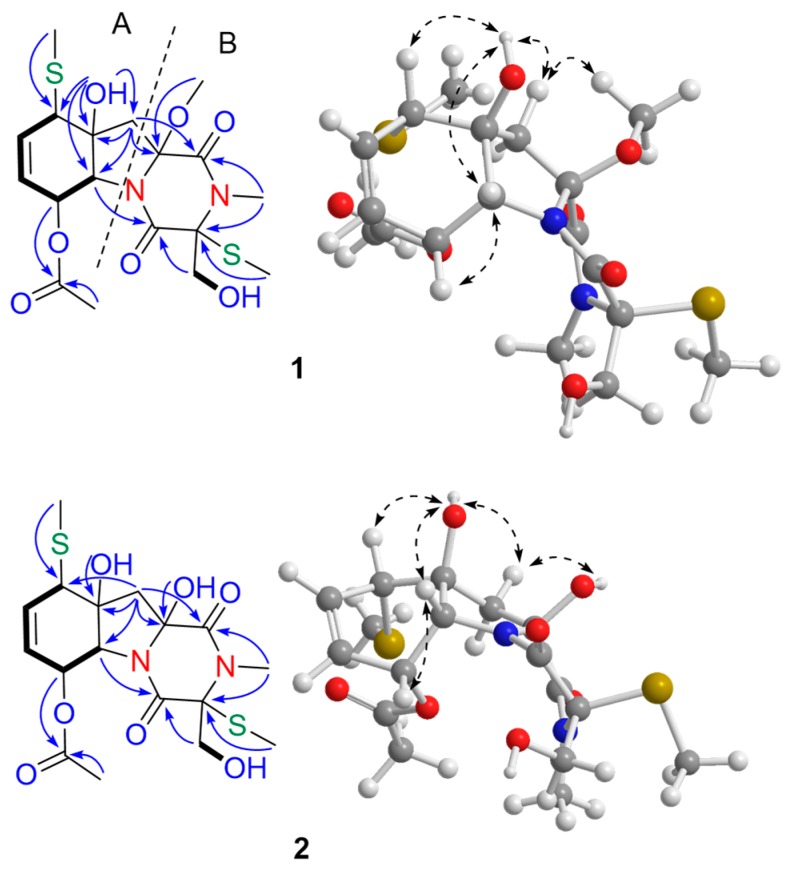
Key COSY (▬), HMBC (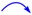), and NOESY (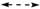) correlations for compounds **1** and **2**.

Compound **1**, a colorless oil, had the molecular formula of C_18_H_26_N_2_O_7_S_2_, as determined by HRESIMS (*m/z* 469.1063 [M + Na]^+^, calcd for 469.1074), corresponding to seven degrees of unsaturation. The ^1^H NMR spectrum revealed the presence of five methyl singlets (*δ*_H_ 3.15, 2.99, 2.26, 2.05, and 1.76), a disubstituted double bond (*δ*_H_ 6.00 (2H, m)), and a series of aliphatic methylene or methine multiplets (5.60 (1H, s), 5.52 (1H, s), 5.48 (1H, d, *J* = 5.6 Hz), 4.23 (1H, d, *J* = 1.8 Hz), 3.93 (1H, dd, *J* = 11.7, 6.7 Hz), 3.68 (1H, m), 3.66 (1H, m), 2.54 (1H, m), and 1.86 (1H, d, *J* = 14.2 Hz)). The ^13^C NMR, in combination with HSQC experiments, resolved 18 carbon resonances attributed to three carbonyl groups (*δ*_C_ 169.4, 167.6, and 163.5), a disubstituted double bond (*δ*_C_ 137.2 and 127.4), three sp^3^ quaternary carbons (*δ*_C_ 90.6, 81.0, and 76.8), three sp^3^ methines (*δ*_C_ 70.5, 65.2, and 51.8), two sp^3^ methylene (*δ*_C_ 62.8 and 42.3), a methoxyl group (*δ*_C_ 52.1), an *N*-methyl group (*δ*_C_ 28.6), and three methyl groups (*δ*_C_ 20.6, 16.1, and 11.0) (Table 1). The 1D NMR data of **1** showed resonance characteristics of a thiodiketopiperazine framework similar to that of **5**, except for the absence of a double bond and presence of a methoxy group. In comparison with **5**, the ^13^C NMR spectroscopic data for **1** differed significantly from C-10 to C-13, with the distinctly upfield-shifted carbon at C-13 (*δ*_C_ 90.6 in **1** and *δ*_C_ 75.2 in **5**) and the downfield-shifted at C-10 and C-11 (*δ*_C_ 51.8 and 81.0 in **1** and *δ*_C_ 120.5 and 135.7 in **5**, respectively). Detailed 2D NMR analyses (^1^H–^1^H COSY, HSQC, and HMBC) allowed the establishment of the gross structure of **1** as depicted in Figure 2. HMBC correlations from H-7 (*δ*_H_ 5.48) and H_3_-18 (*δ*_H_ 2.05) to C-17 (*δ*_C_ 169.4), from H_3_-19 (*δ*_H_ 2.26) to C-10 (*δ*_C_ 51.8), and from OH-11 (*δ*_H_ 5.60) to C-6/C-10/C-11/C-12 (*δ*_C_ 70.5, 51.8, 81.0, and 42.3, respectively), and ^1^H–^1^H COSY correlations of H-6/H-7/H-8/H-9/H-10 confirmed the presence of fragment A (Figure 2). Fragment B was very similar to **5**, except for the distinct downfield-shifted of the O-methyl group when compared with the S-methyl group (*δ*_C_ 52.1 in **1** and *δ*_C_ 15.1 in **5**). The methyl group was assigned to C-13 by the HMBC correlations from the methyl group (*δ*_H_ 3.15) to the severely downfield-shifted C-13 (90.6 ppm in **1** and 75.2 ppm in **5**, respectively).

The relative configuration of **1** was established on the basis of the interpretation of the NOESY data and ^1^H−^1^H coupling constants (Figure 2 and Table 1). The strong NOE interactions of H-10/OH-11, OH-11/H-6, H-6/H-7, OH-11/H-12β, and H-12β/H_3_-20 indicated that H-10, OH-11, H-6, H-7, and CH_3_-20 occupied the axial bonds of the cyclohexane-ring portion in a chair conformation and were arbitrarily assigned *β*-orientations. Additionally, the axial orientation of both H-6 and H-7 were in good accordance with their small coupling constant of 1.8 Hz.

**Figure 3:**
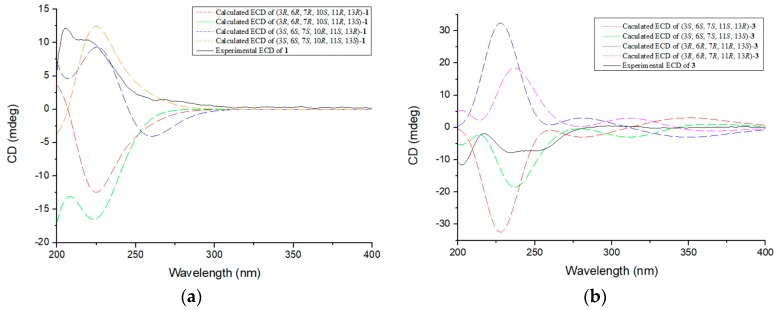
(**a**) Experimental electronic circular dichroism (ECD) spectra of geospallin A (**1**) in MeOH and calculated ECD spectra of (3*R*, 6*R*, 7*R*, 10*S*, 11*R*, 13*R*)-**1**, (3*R*, 6*R*, 7*R*, 10*S*, 11*R*, 13*S*)-**1**, (3*S*, 6*S*, 7*S*, 10*R*, 11*S*, 13*R*)-**1**, and (3*S*, 6*S*, 7*S*, 10*R*, 11*S*, 13*S*)-**1**; (**b**) Experimental ECD spectra of geospallin C (**3**) in MeOH and calculated ECD spectra of (3*S*, 6*S*, 7*S*, 11*S*, 13*R*)-**3**, (3*S*, 6*S*, 7*S*, 11*S*, 13*S*)-**3**, (3*R*, 6*R*, 7*R*, 11*R*, 13*S*)-**3**, and (3*R*, 6*R*, 7*R*, 11*R*, 13*R*)-**3**. The calculated ECD spectra were computed at the B3LYP/6-311G (2d+p) level.

In order to define the absolute configuration of **1**, the ECD spectrum of (3*R*, 6*R*, 7*R*, 10*S*, 11*R*, 13*R*)-**1**, (3*R*, 6*R*, 7*R*, 10*S*, 11*R*, 13*S*)-**1**, (3*S*, 6*S*, 7*S*, 10*R*, 11*S*, 13*R*)-**1**, and (3*S*, 6*S*, 7*S*, 10*R*, 11*S*, 13*S*)-**1** were calculated by the time-dependent density functional theory (TDDFT) computational method and compared with the experimental spectra of **1** (for details of calculations, see Supplementary Materials). The experimental ECD spectrum of **1** showed an ECD curve with positive Cotton effects around 219 nm (Figure 3a). The calculated ECD spectrum for (3*S*, 6*S*, 7*S*, 10*R*, 11*S*, 13*S*)-**1** showed a similar ECD curve with positive Cotton effects at 220 nm, indicating that **1** had an (3*S*, 6*S*, 7*S*, 10*R*, 11*S*, 13*S*)-configuration. Compound **1** was given the trivial name geospallin A.

The HRESIMS data of **2** exhibited a sodium adduct ion at *m/z* 455.0907 [M + Na]^+^ (calcd 455.0917), consistent with the molecular formula C_17_H_24_N_2_O_7_S_2_Na, showing 14 mass units less than that of **1**. The 1D NMR data of **2** were similar to those of **1**, except for the absence of a methoxy group in **2**, indicating **2** was a demethylated derivative of **1**. The additional hydroxy group was located at C-13 by HMBC correlation from the hydroxyl group (*δ*_H_ 6.04) to C-13 (*δ*_C_ 86.3) (Figure 2). The absolute configuration of **2** was confirmed by using the same methods as described for **1.** The experimental ECD spectrum for **2** showed a similar ECD curve with positive Cotton effects at 220 nm (Figure S46 in Supplementary Materials), indicating that compound **2** has a (3*S*, 6*S*, 7*S*, 10*R*, 11*S*, 13*S*)-configuration. Compound **2** was given the trivial name geospallin B. 

**Figure 4:**
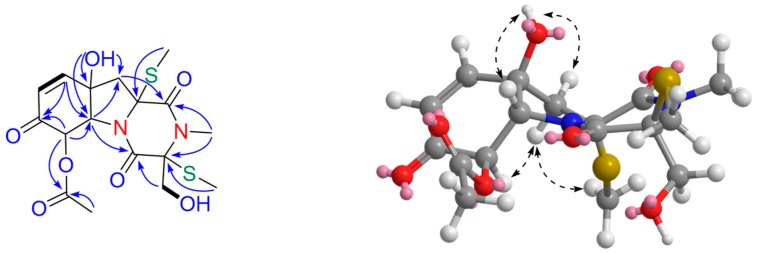
Key ^1^H-^1^H COSY (▬), HMBC (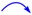), and NOESY (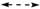) correlations for geospallin C (**3**).

Compound **3**, a colorless oil, had the molecular formula of C_17_H_22_N_2_O_7_S_2_, as determined by HRESIMS (*m/z* 453.0764 [M + Na]^+^, C_17_H_22_N_2_O_7_S_2_Na, calcd for 453.0761), corresponding to eight degrees of unsaturation. The ^1^H and ^13^C NMR spectra (Table 1) of **3** showed high similarity to those of **5**, except for the presence of an *α*,*β*-unsaturated ketone moiety (*δ*_C_ 191.6, 125.4, and 150.5) in **3** instead of two double bonds in **5**, indicating that **3** was an oxidative derivative of **5**. This was supported by detailed 2D NMR spectra analyses (Figure 4). The location of the *α*,*β*-unsaturated ketone moiety was assigned at C-11 by HMBC correlations from OH-11 (*δ*_H_ 6.00) to C-10, C-11, C-6, and C-12 (*δ*_C_ 150.5, 75.1, 69.8, and 49.3, respectively); from H-10 (*δ*_H_ 6.98) to C-6 and C-8 (*δ*_C_ 191.6); and from H-7 (*δ*_H_ 5.80) to the carbonyl group (*δ*_C_ 191.6, C-8) and acetyl group (*δ*_C_ 168.9). The relative configuration of **3** was deduced by NOESY correlations of H-6/H-12*β*, H-7/H12*α*, and H-12*α*/SMe-13.

The absolute structure of **3** was also deduced by comparison of the experimental and calculated ECD spectra generated by TDDFT calculations in the Gaussian 16 program (Figure 3b). As illustrated in Figure 3b, the experimentally acquired ECD spectrum for **3** agreed well with the ECD curve computed for (3*S*, 6*S*, 7*S*, 11*S*, 13*S*)-**3**. Compound **3** was given the trivial name geospallin C.

### 2.2. Angiotensin-Converting Enzyme (ACE) Inhibitory Assay

The twelve thiodiketopiperazines (**1**−**12**) reported in this study were produced in sufficient amounts to allow testing for the inhibition of angiotensin-converting enzyme (Table 2). IC_50_ values above 100 μM were not determined, while compounds **1**−**3** showed inhibitory activity against ACE with an IC_50_ value range of 29−35 μM.

### 2.3. α-Glucosidase Inhibitory Activity Assay

All compounds were evaluated in vitro for *α*-glucosidase inhibitory activity. However, none of the compounds showed inhibitory activity against *α*-glucosidase at a concentration of 100 μM.

## 3. Materials and Methods

### 3.1. General Experimental Procedures

IR spectra were carried out on a Shimadzu IR Affinity-1 spectrophotometer (Shimadzu Corporation, Kyoto, Japan). UV data was acquired using a Shimadzu UV-2600 spectrophotometer (Shimadzu Corporation, Kyoto, Japan). Optical rotations were obtained on an Anton Paar MCP-500 (Anton Paar, Graz, Austria). Circular dichroism (CD) spectra were recorded on a Jasco 820 spectropolarimeter (Jasco Corporation, Kyoto, Japan). NMR spectra were determined on a Bruker Avance-400 spectrometer (Bruker Corporation, Fremont, CA, USA). ESI-MS spectra were measured on an Agilent Technologies 1290-6430A Triple Quad LC/MS (Agilent Technologies Inc., Santa Clara, CA, USA), and HRESIMS was measured on a Thermo MAT95XP high-resolution mass spectrometer (Thermo Fisher Scientific, Bremen, Germany). A Shimadzu LC-20 AP (Shimadzu Corporation, Kyoto, Japan) equipped with an SPD-M20A Photo-Diode Array (PDA) detector (Shimadzu Corporation, Kyoto, Japan) was used for HPLC analysis. A YMC-pack ODS-A column (250 × 20 mm, 5 μm, 12 nm, YMC CO., Ltd., Kyoto, Japan) was used for preparative HPLC separation. Column chromatography was conducted using a commercial silica gel (SiO_2_; 200–300 mesh; Qingdao Haiyang Chemical Co. Ltd., Qingdao, China) and Sephadex LH-20 gel (Amersham Biosciences, Uppsala, Sweden). All solvents were of analytical grade (Guangzhou Chemical Regents Company, Ltd., Guangzhou, China).

### 3.2. Fungal Material and Identification

The fungal strain FS140 was isolated from a deep-sea sludge in the South China Sea (19°28.581′ N, 115°27.251′ E, depth 2403 m) in September 2011. The isolate was identified as *Geosmithia pallida* FS140 by sequence analysis of the internal transcribed spacer (ITS) region of the ribosomal DNA. The sequence data have been submitted to GenBank (accession no. MK047400), and FS140 has 99% similarity with *Geosmithia pallida* CCF4279 (accession no. KF808303). The strain was deposited in the Guangdong Provincial Key Laboratory of Microbial Culture Collection and Application, Guangdong Institute of Microbiology, Guangzhou, People’s Republic of China. Working stocks were prepared on potato dextrose agar (PDA) slants and stored at 4 °C.

### 3.3. Fermentation, Extraction, and Isolation

Three pieces (0.5 × 0.5 cm^2^) of mycelial agar plugs of *G. pallida* FS140 were inoculated into 250 mL of PD medium (potato 200 g/L, glucose 20 g/L, KH_2_PO_4_ 3 g/L, MgSO_4_·7H_2_O 1.5 g/L, vitamin B_1_ 10 mg/L, sea salt 15 g/L) in 500 mL Erlenmeyer flasks and incubated for 2 days in a rotary shaker (200 r/m) at 28 °C. The seed cultures (10%) were then aseptically transferred into 500 mL of PD medium in 1000 mL Erlenmeyer flasks and kept shaking (120 r/m) at 28 °C for 7 days. The whole fermentation broth (80 L) was filtered through cheese cloth to separate the supernatant from the mycelia. The supernatant was extracted with EtOAc (4 × 25 L) and evaporated under reduced pressure to give a dark brown oily residue (31 g). The EtOAc-soluble fraction was separated over a column of silica gel and eluted with petroleum ether/EtOAc in a linear gradient (30:1 → 1:1) and followed by CHCl_3_/MeOH in linear gradient (10:1 → 0:1) to give 18 fractions (F1–F18). F7 was separated on a preparative reversed-phase (RP) HPLC system equipped with a C-18 column (YMC*GEL ODS-A, 120A S-5 μm, 250 × 20 mm, MeOH/H_2_O, 0.1:0.9 → 1.0:0, 10 mL/min) to give 25 fractions (F7a–F7y). F7c was chromatographed over a Sephadex LH-20 column eluted with CHCl_3_/MeOH (1:1, *v*/*v*), then further separated by preparative RP HPLC on the ODS-A column (MeCN/H_2_O, 40:60, 10 mL/min) to yield **2** (4.5 mg, *t*_R_ = 16.0 min), **9** (23.0 mg, *t*_R_ = 25.0 min), **10** (6.1 mg, *t*_R_ = 31.0 min), and **8** (7.6 mg, *t*_R_ = 34 min), sequentially. F7f was successfully separated by preparative RP HPLC on the ODS-A column (MeCN/H_2_O, 45:55, 10 mL/min) to afford **1** (5.9 mg, *t_R_* = 12.1 min) and **3** (6.8 mg, *t_R_* = 13.4 min). F7k was separated on a preparative RP HPLC on the ODS-A column (MeCN/H_2_O, 50:50, 10 mL/min) to yield **4** (427 mg), and F7m was purified by column chromatography on a Sephadex LH-20 (CHCl_3_/MeOH, 1:1, *v*/*v*) to afford **5** (312 mg). F9 was purified by preparative HPLC on the ODS-A column (MeCN/H_2_O, 60:40, 10 mL/min) to give **7** (28 mg, *t*_R_ = 14.2 min) and **12** (16.8 mg, *t*_R_ = 19.3 min), while F10 was subjected to a Sephadex LH-20 column elution with CHCl_3_/MeOH (1:1), then further separated by preparative HPLC on the ODS-A column (MeCN/H_2_O, 65:35, 10 mL/min) to obtain **6** (39 mg, *t*_R_ = 9.6 min) and **11** (18.1 mg, *t*_R_ = 11.8 min), successively.

Geospallin A (**1**): colorless oil; [α ${]}_{D}^{25}$ +87.7 (*c* 0.1, MeOH); CD (MeOH, *c* 0.001 mg/mL) 219 nm (∆ε + 3.16); IR ν_max_ 3443, 2924, 1739, 1662, 1372, 1237, 1095, 1022 cm^−1^; ^1^H and ^13^C NMR data, see Table 1; HRESIMS *m/z* 469.1063 ([M + Na]^+^, calcd for 469.1074).

Geospallin B (**2**): colorless oil; [α ${]}_{D}^{25}$ +84.9 (*c* 0.1, MeOH); CD (MeOH, *c* 0.001 mg/mL) 225 nm (∆ε + 3.21); IR ν_max_ 3315, 3056, 2923, 1738, 1667, 1422, 1373, 1265, 1236 cm^−1^; ^1^H and ^13^C NMR data, see Table 1; HRESIMS *m/z* 455.0907 ([M + Na]^+^, calcd for 455.0917).

Geospallin C (**3**): colorless oil; [α ${]}_{D}^{25}$ −28.2 (*c* 0.1, MeOH); CD (MeOH, *c* 0.001 mg/mL) 202 (∆*ε* − 3.52), 234 (∆*ε* − 2.36) nm; UV (MeOH) λ_max_ (log ε) 210 (4.26) nm; IR ν_max_ 3407, 2925, 1749, 1703, 1654, 1415, 1376, 1222, 1085, 1038 cm^−1^; ^1^H and ^13^C NMR data, see Table 1; HRESIMS *m/z* 453.0764 ([M + Na]^+^, calcd for 453.0761).

### 3.4. Quantum Chemical ECD Calculation

The quantum chemical ECD calculation methods were used to establish the absolute configurations of compounds **1**–**3**. The 3D structures were generated by Discover Studio 2.5. The conformational search was performed by the Conformer Searching module of Open Babel 2.4.1 using a genetic algorithm and the MMFF94 molecular mechanics force field. The geometry optimizations were then performed by using density functional theory (DFT) at the b3lyp/6-311+g(2d,p) level. These stable conformers, which had no imaginary frequency, were subsequently submitted to ECD calculations by TDDFT calculations at the b3lyp/6-311+g(2d,p) level. The solvent effects were taken into account by the integral equation formalism polarizable continuum model (IEFPCM, methanol). All calculations were performed with the Gaussian 16 A.03 program [18]. The calculated spectra were drawn using SpecDis software with a UV shift to the ECD spectra.

### 3.5. Angiotensin-Converting Enzyme (ACE) Inhibitory Assay

ACE inhibitory activity was determined by a previously reported method [19]. In brief, 20 μL thiodiketopiperazines dissolved in DMSO with different concentrations were added to 120 μL N-hippuryl–His–Leu substrates, then preheated in water for 3–5 min; next, 10 μL ACE enzymatic solution was added and mixtures were incubated at 37 °C for 60 min, and 150 μL 1 M HCl was added to stop the reaction. The mixture was loaded for HPLC with a flow rate of 0.5 mL/min by 60% methanol, and the absorbance at 228 nm was detected. To serve as a blank, 10 μL pH 8.3 boric acid replaced the thiodiketopiperazines. Hippuric acid solutions at 10, 20, 40, 60, 80, and 100 μg/mL were prepared using 10 μL pH 8.3 boric acid. One enzymatic unit was defined as the amount giving the production of 1 μM hippuric acid by the catalyzation of the substrate N-hippuryl–His–Leu at 37 °C in 1 min. Captopril was used as a positive control. Physiological saline with a concentration of 0.9% (*w*/*w*) was used as a negative control in our experiment, which exhibited no inhibitory activity towards ACE. The IC_50_ values of thiodiketopiperazines were calculated after the ACE inhibitory experiments were conducted, using different concentrations of thiodiketopiperazines. 

### 3.6. α-Glucosidase Inhibitory Activity Assay

An assay of α-glucosidase inhibitory activity was performed as previously described [20].

## 4. Conclusions

In this study, twelve diketopiperazines, including three new thiodiketopiperazines, were isolated from the deep-sea-derived fungus *Geosmithia pallida*. All the stereochemical configurations of the new compounds, including their absolute configurations, were established. Geospallins A and B (**1** and **2**) represent rare examples of thiodiketopiperazines featuring an S-methyl group at C-10 and a tertiary hydroxyl group at C-11, and their *S*-configuration at both C-7 and C-13 is the first report of such. These thiodiketopiperazines were examined for their angiotensin-converting enzyme inhibitory assay, and geospallins A–C (**1**–**3**) showed inhibitory activity, with IC_50_ values of 29–35 µM.

## Figures and Tables

**Table 1:** ^1^H (400 MHz) and ^13^C (100 MHz) NMR data for compounds **1**–**3** (in DMSO-*d_6_*)^a^.

No.	1		2		3	
	^1^H	^13^C	^1^H	^13^C	^1^H	^13^C
1		163.5		166.1		165.0
3		76.8		77.9		70.8
4		167.6		167.6		162.6
6	4.23, d (1.8)	70.5	4.32, d (1.8)	70.5	4.96, d (11.0)	69.8
7	5.48, d (5.6)	65.2	5.32, d (3.2)	66.4	5.80, d (11.0)	75.1
8	6.00, m	127.4	5.96, d (3.2)	127.4		191.6
9	6.00, m	137.2	5.96, d (3.2)	137.2	6.09, d (10.3)	125.4
10	3.66, m	51.8	3.65, s	51.3	6.98, d (10.3)	150.5
11		81.0		81.4		75.1
12	2.54, m 1.86, d (14.2)	42.3	2.28, d (8.5) 2.12, d (14.2)	45.6	3.07, d (14.9) 2.95, d (14.9)	49.3
13		90.6		86.3		69.1
14	3.93, dd (11.7, 6.7) 3.68, m	62.8	3.81, m 3.71, dd (10.9, 3.6)	62.2	4.18, dd (11.5, 6.0) 3.74, dd (11.5, 4.7)	62.6
N-Me	2.99, s	28.6	2.99, s	29.1	2.99, s	28.8
SMe-3	1.76, s	11.0	1.80, d (3.6)	10.6	2.16, s	13.0
OAc-7		169.4		169.4		168.9
	2.05, s	20.6	2.05, s	20.6	2.07, s	20.4
SMe-10	2.26, s	16.1	2.26, s	16.1		
OMe-13	3.15, s	52.1			2.18, s	14.6
11-OH	5.60, s		5.68, s		6.00, s	
13-OH			6.04, brs			
14-OH	5.52, s		6.64, brs		5.34, t (5.4)	

^a^ Chemical shifts are in ppm; coupling constant *J* is in Hz.

**Table 2:** IC_50_ values of compounds **1**–**3** against angiotensin-converting enzyme (ACE).

Compounds	IC_50_ (µM)
**1**	35 ± 5.2
**2**	31 ± 3.3
**3**	29 ± 3.3
**Captopril**	0.041 ± 0.005

## Supplementary Materials

The following is available online at http://www.mdpi.com/1660-3397/16/12/464/s1, The ^1^H- and ^13^C-NMR data of **1**–**12** and the HRESIMS and 2D-NMR spectra of compounds **1**–**3** (Figures S1–S47).
