# Supplementary material for: Geospallins A–C: New Thiodiketopiperazines with Inhibitory Activity against Angiotensin-Converting Enzyme from a Deep-Sea-Derived Fungus Geosmithia pallida FS140
Source: Mar Drugs. 2018 Nov 23;16(12):464. doi: 10.3390/md16120464 (PMC6315564; doi:10.3390/md16120464)
Supplement: Supplementary file 1 [file marinedrugs-16-00464-s001.pdf]

Supplementary Materials

Geospallins A–C: New Thiodiketopiperazines with Inhibitory Activity against Angiotensin-Converting Enzyme from a Deep-Sea-Derived Fungus *Geosmithia pallida* FS140

Figure S1. ^{1}H NMR spectrum of geospallin A (**1**) in DMSO-d_6.

Figure S2. ^{13}C NMR spectrum of geospallin A (**1**) in DMSO-d_6.

Figure S3. HSQC spectrum of geospallin A (**1**) in DMSO-d_6.

Figure S4. ^{1}H–^{1}H COSY spectrum of geospallin A (**1**) in DMSO-d_6.

Figure S5. HMBC spectrum of geospallin A (**1**) in DMSO-d_6.

Figure S6. NOESY spectrum of geospallin A (**1**) in DMSO-d_6.

Figure S7. HRESIMS spectrum of geospallin A (**1**).

Figure S8. ^{1}H NMR spectrum of geospallin B (**2**) in DMSO-d_6.

Figure S9. ^{13}C NMR spectrum of geospallin B (**2**) in DMSO-d_6.

Figure S10. HSQC spectrum of geospallin B (**2**) in DMSO-d_6.

Figure S11. ^{1}H–^{1}H COSY spectrum of geospallin B (**2**) in DMSO-d_6.

Figure S12. HMBC spectrum of geospallin B (**2**) in DMSO-d_6.

Figure S13. NOESY spectrum of geospallin B (**2**) in DMSO-d_6.

Figure S14. HRESIMS spectrum of geospallin B (**2**).

Figure S15. ^{1}H NMR spectrum of geospallin C (**3**) in DMSO-d_6.

Figure S16. ^{13}C NMR spectrum of geospallin C (**3**) in DMSO-d_6.

Figure S17. HSQC spectrum of geospallin C (**3**) in DMSO-d_6.

Figure S18. ^{1}H–^{1}H COSY spectrum of geospallin C (**3**) in DMSO-d_6.

Figure S19. HMBC spectrum of geospallin C (**3**) in DMSO-d_6.

Figure S20. NOESY spectrum of geospallin C (**3**) in DMSO-d_6.

Figure S21. HRESIMS spectrum of geospallin C (**3**).

Figure S22. ^{1}H NMR spectrum of bisdethiobis (methylthio)gliotoxin (**4**) in CD_3OD.

Figure S23. ^{13}C NMR spectrum of bisdethiobis (methylthio)gliotoxin (**4**) in CD_3OD.

Figure S24. ^{1}H NMR spectrum of 6-acetylbis(methylthio)gliotoxin (**5**) in CD_3OD.

Figure S25. ^{13}C NMR spectrum of 6-acetylbis(methylthio)gliotoxin (**5**) in CD_3OD.

Figure S26. ^{1}H NMR spectrum of 6-deoxy-5a,6-didehydrogliotoxin (**6**) in DMSO-d_6.

Figure S27. ^{13}C NMR spectrum of 6-deoxy-5a,6-didehydrogliotoxin (**6**) in DMSO-d_6.

Figure S28. ^{1}H NMR spectrum of 5a,6-didehydrogliotoxin (**7**) in DMSO-d_6.

Figure S29. ^{13}C NMR spectrum of 5a,6-didehydrogliotoxin (**7**) in DMSO-d_6.

Figure S30. ^{1}H NMR spectrum of 6-(phenylmethyl)-(3R,6R)-2,5-piperazinedione (**8**) in CD_3OD.

Figure S31. ^{13}C NMR spectrum of 6-(phenylmethyl)-(3R,6R)-2,5-piperazinedione (**8**) in CD_3OD.

Figure S32. ^{1}H NMR spectrum of 3-(hydroxymethyl)-3,6-bis(methylthio)-6-(phenylmethyl)-(3R,6R)-2,5-piperazinedione (**9**) in DMSO-d_6.

Figure S33. ^{13}C NMR spectrum of 3-(hydroxymethyl)-3,6-bis(methylthio)-6-(phenylmethyl)-(3R,6R)-2,5-piperazinedione (**9**) in DMSO-d_6.

Figure S34. ^{1}H NMR spectrum of 3-(hydroxymethyl)-6-(methoxyl)-6-(phenylmethyl) -(3R,6R)-2,5-piperazinedione (**10**) in CD_3OD.

Figure S35. ^{13}C NMR spectrum of 3-(hydroxymethyl)-6-(methoxyl)-6-(phenylmethyl) -(3R,6R)-2,5-piperazinedione (**10**) in CD_3OD.

Figure S36. ^{1}H NMR spectrum of 5a,6-anhydrobisdethiobis(methylthio)gliotoxin (**11**) in CD_3OD.

Figure S37. ^{13}C NMR spectrum of 5a,6-anhydrobisdethiobis(methylthio)gliotoxin (**11**) in CD_3OD.

Figure S38. ^{1}H NMR spectrum of bisdethiobis (methylthio)gliotoxin (**12**) in CD_3OD.

Figure S39. ^{13}C NMR spectrum of bisdethiobis (methylthio)gliotoxin (**12**) in CD_3OD.

Figure S40. CD spectrum of geospallin A (**1**).

Figure S41. CD spectrum of geospallin B (**2**).

Figure S42. CD spectrum of geospallin C (**3**).

Figure S43. $ESI^{(+)}$MS spectrum of geospallin A (**1**).

Figure S44. $ESI^{(-)}$MS spectrum of geospallin B (**2**).

Figure S45. $ESI^{(-)}$MS spectrum of geospallin C (**3**).

Figure S46. Experimental ECD spectra of geospallin B (**2**) in MeOH and calculated ECD spectra of (3*R*, 6*R*, 7*R*, 10*S*, 11*R*, 13*R*)-**2**, (3*R*, 6*R*, 7*R*, 10*S*, 11*R*, 13*S*)-**2**, (3*S*, 6*S*, 7*S*, 10*R*, 11*S*, 13*R*)-**2**, and (3*S*, 6*S*, 7*S*, 10*R*, 11*S*, 13*S*)-**2**.

Figure S47. ECD spectrum calculation.

Figure S1. ^{1}H NMR spectrum of geospallin A (**1**) in DMSO-d_6.

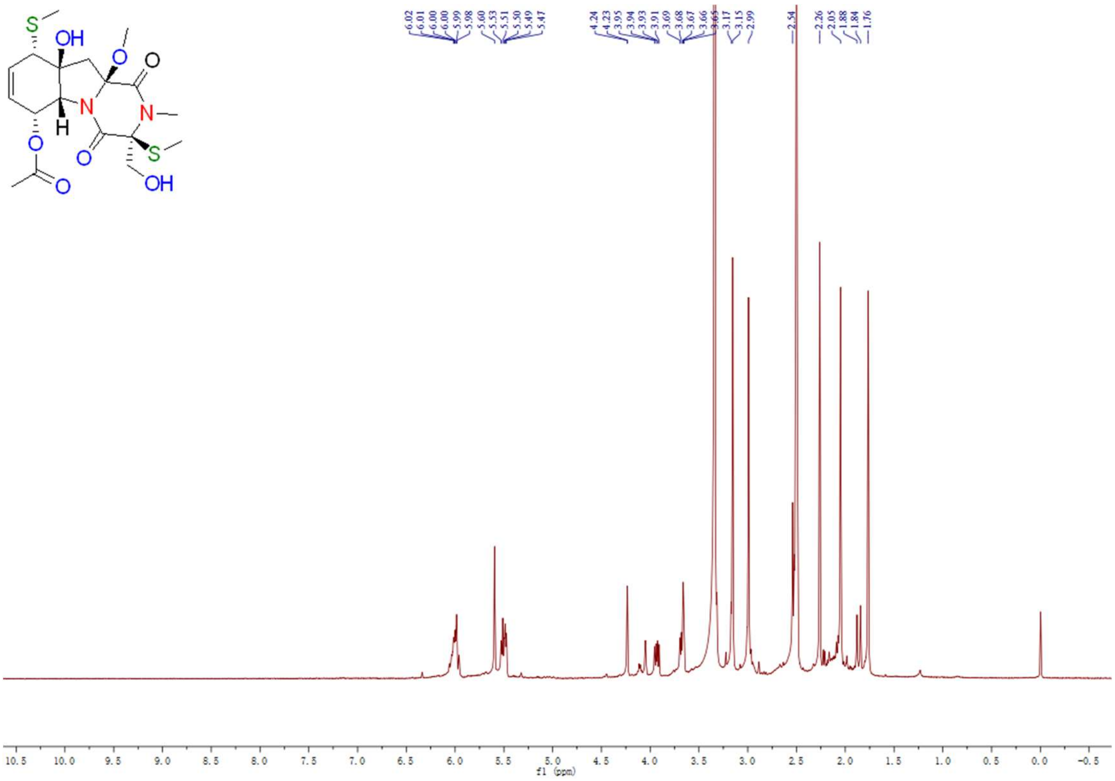

Figure S2. ^{13}C NMR spectrum of geospallin A (**1**) in DMSO-d_6.

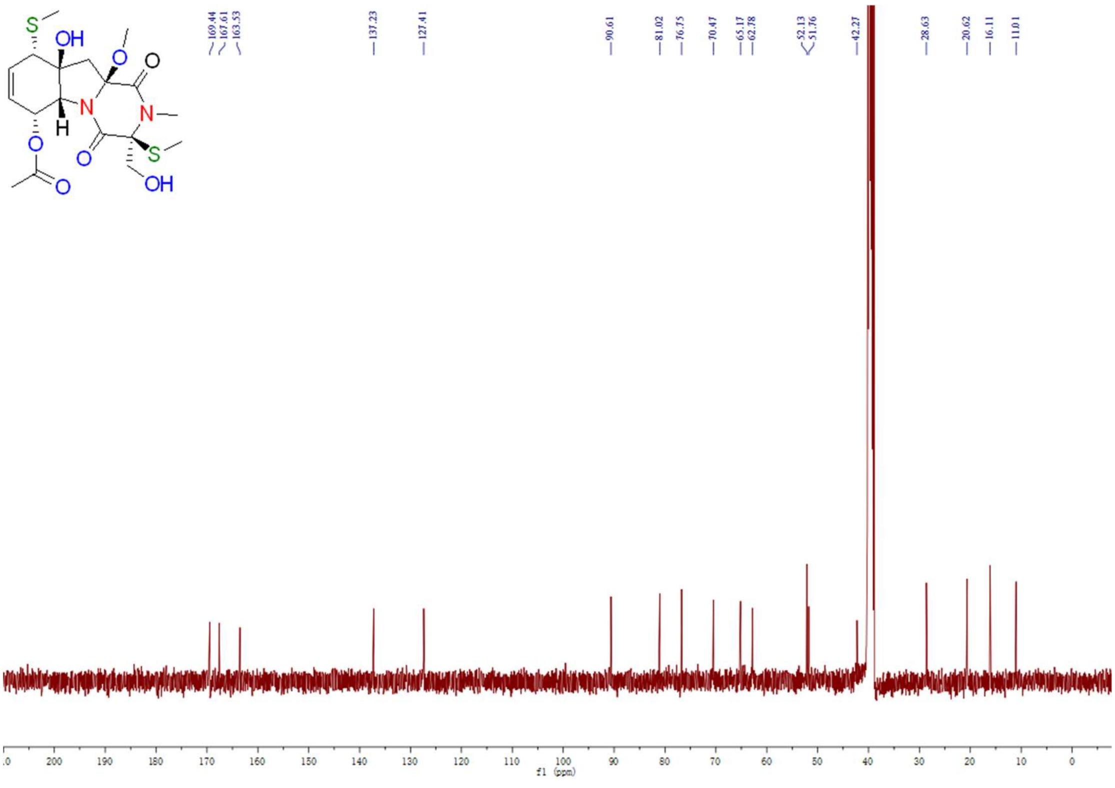

Figure S3. HSQC spectrum of geospallin A (**1**) in DMSO-d_6.

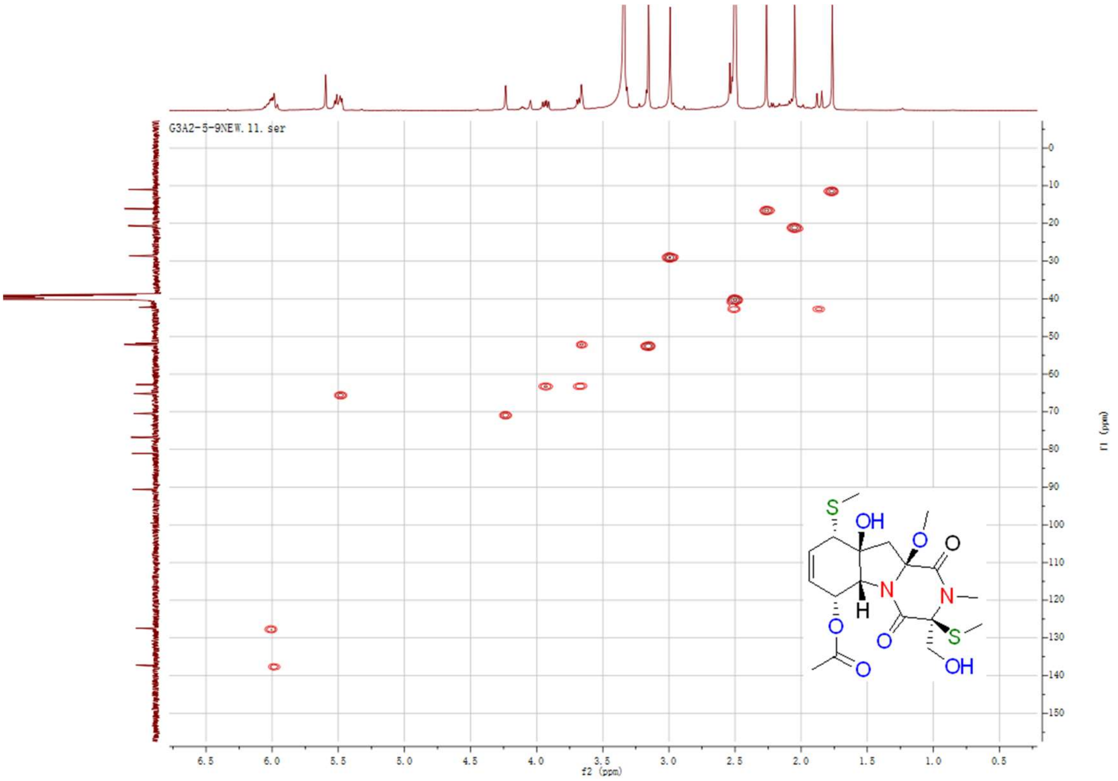

Figure S4. ^1H–^1H COSY spectrum of geospallin A (**1**) in DMSO-d_6.

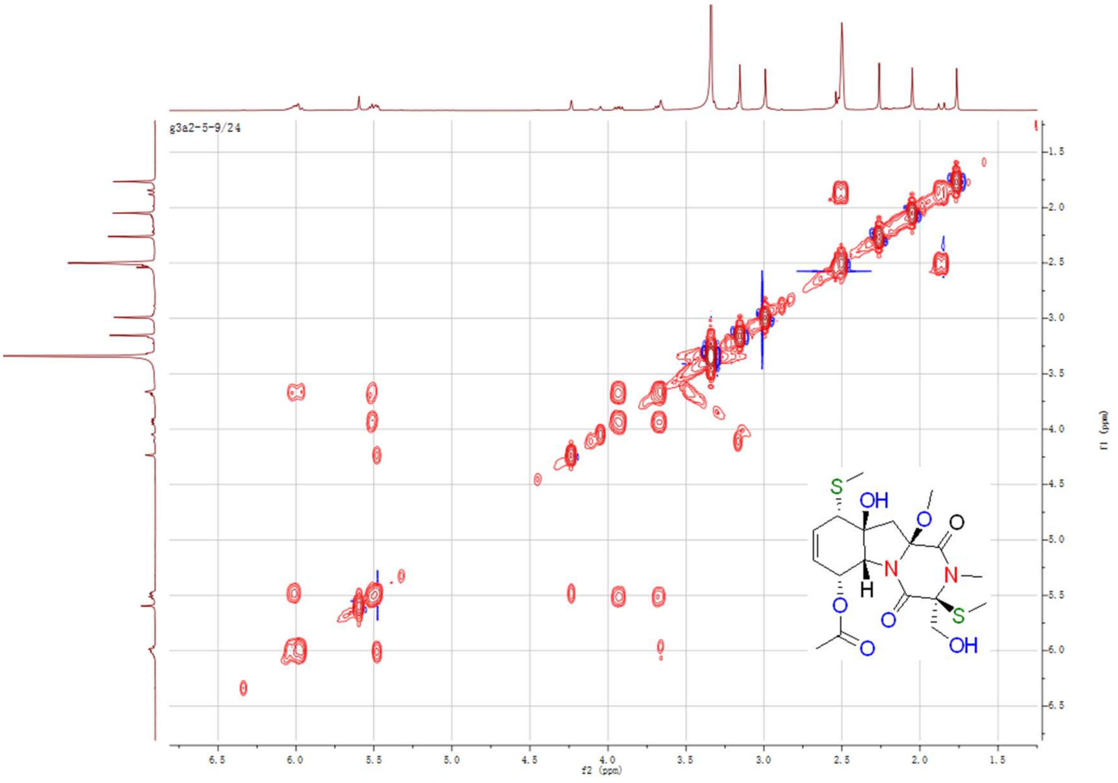

Figure S5. HMBC spectrum of geospallin A (**1**) in DMSO-d_6.

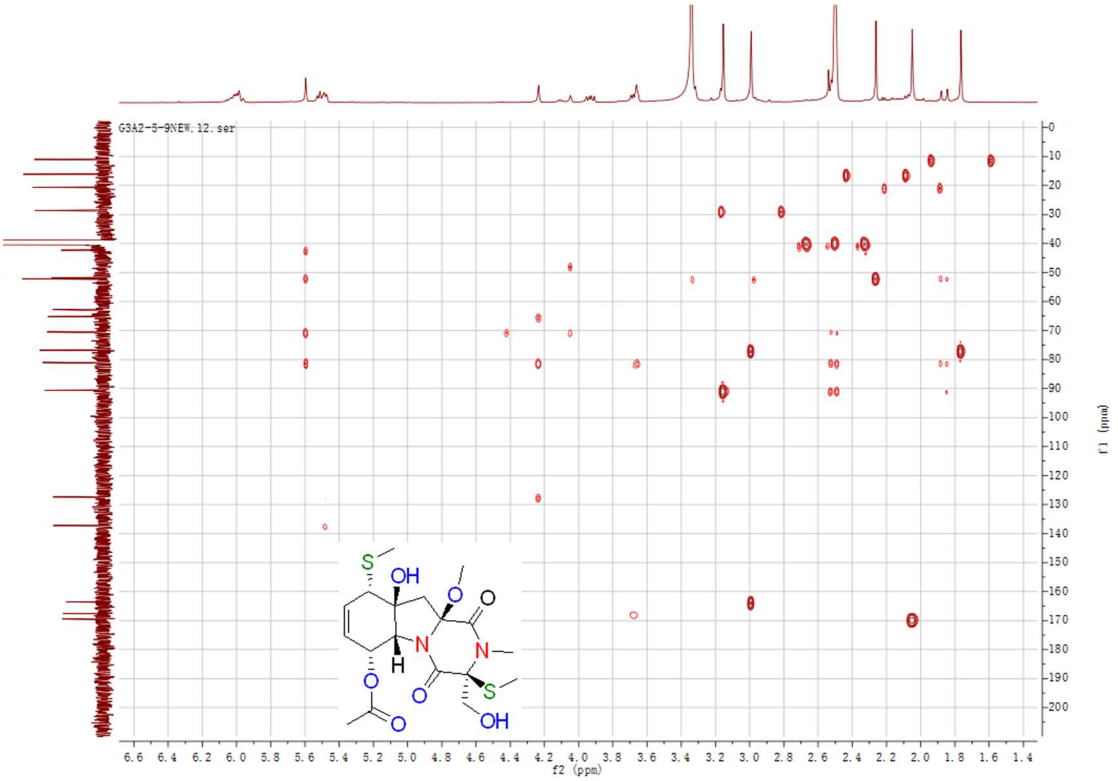

Figure S6. NOESY spectrum of geospallin A (**1**) in DMSO-d_6.

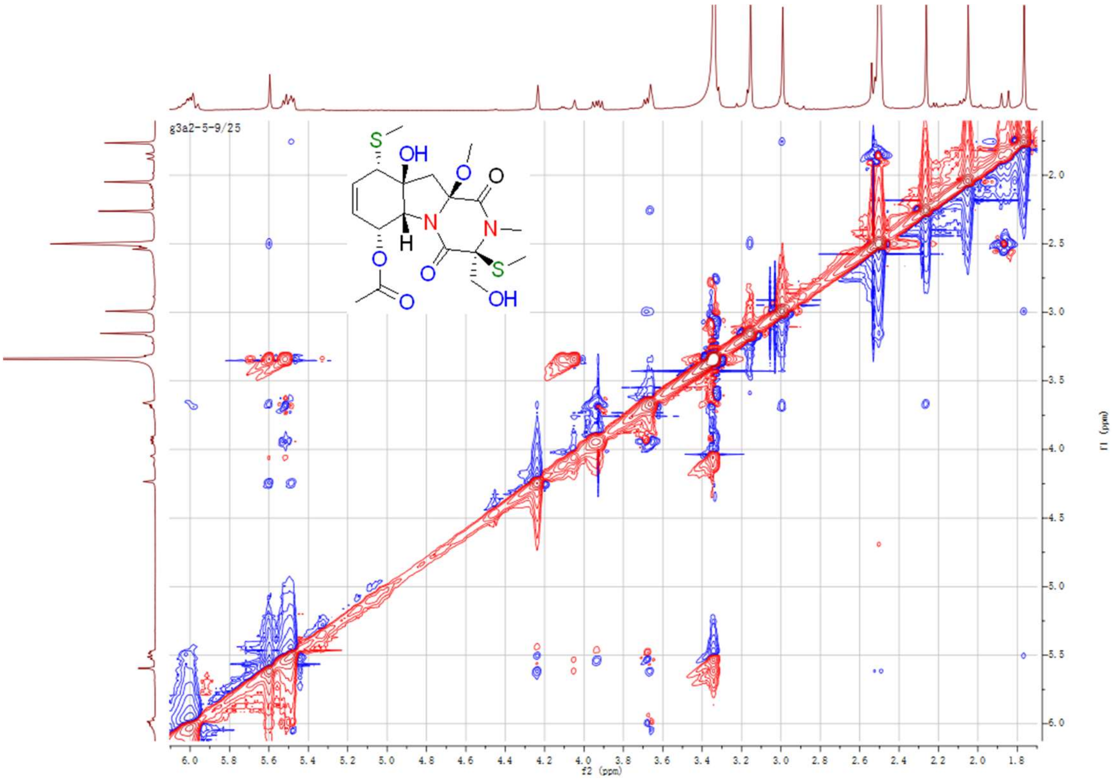

Figure S7. HRESIMS spectrum of geospallin A (**1**).

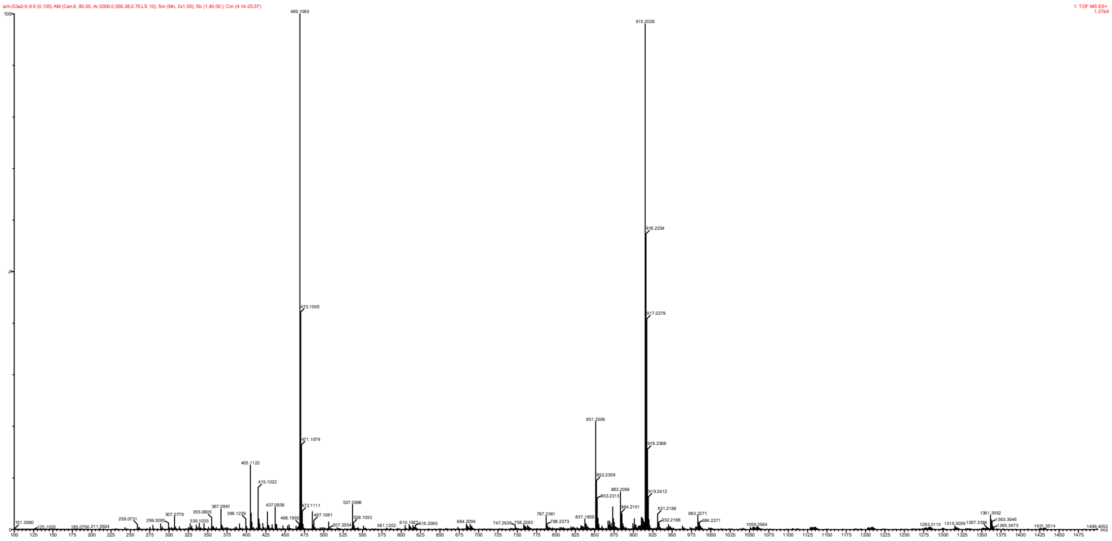

Figure S8. ^{1}H NMR spectrum of geospallin B (**2**) in DMSO-d_6.

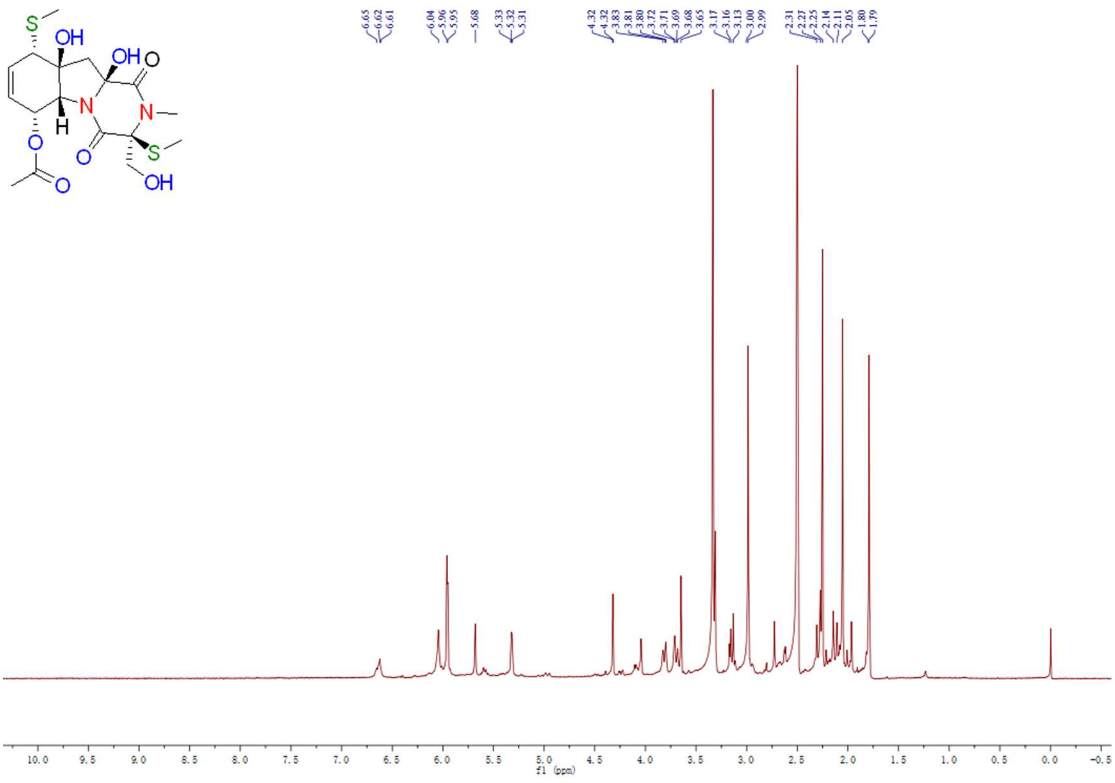

Figure S9. ^{13}C NMR spectrum of geospallin B (**2**) in DMSO-d_6.

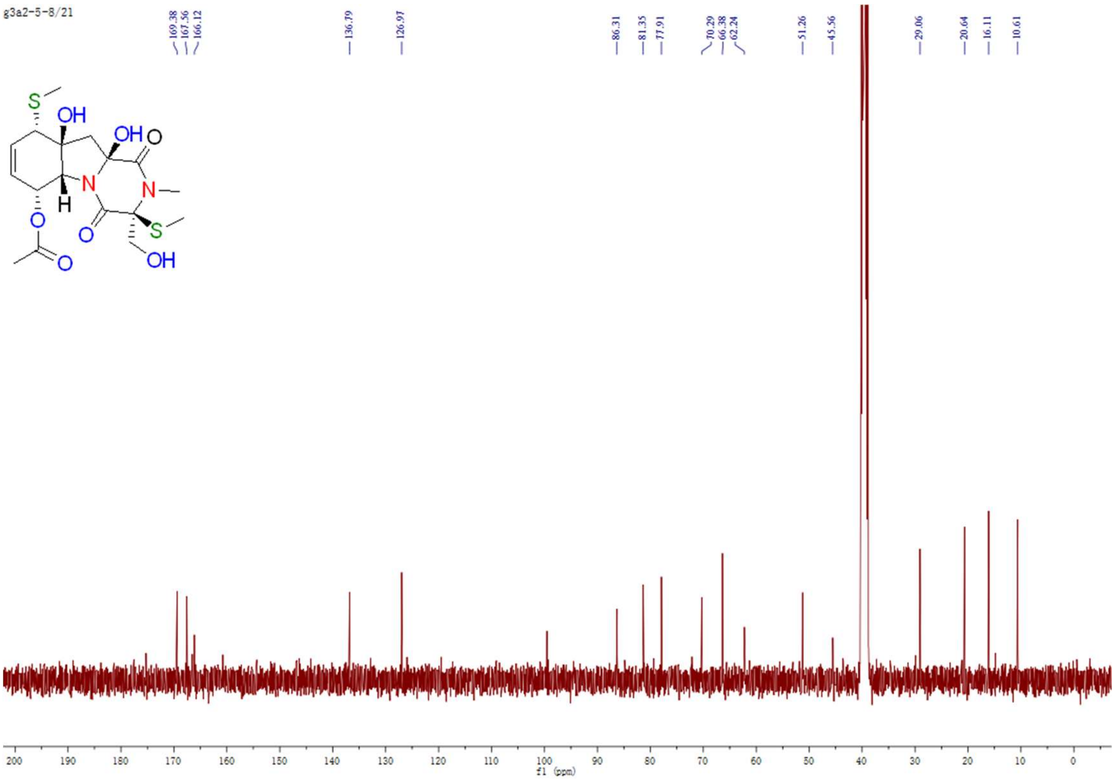

Figure S10. HSQC spectrum of geospallin B (**2**) in DMSO-d_6.

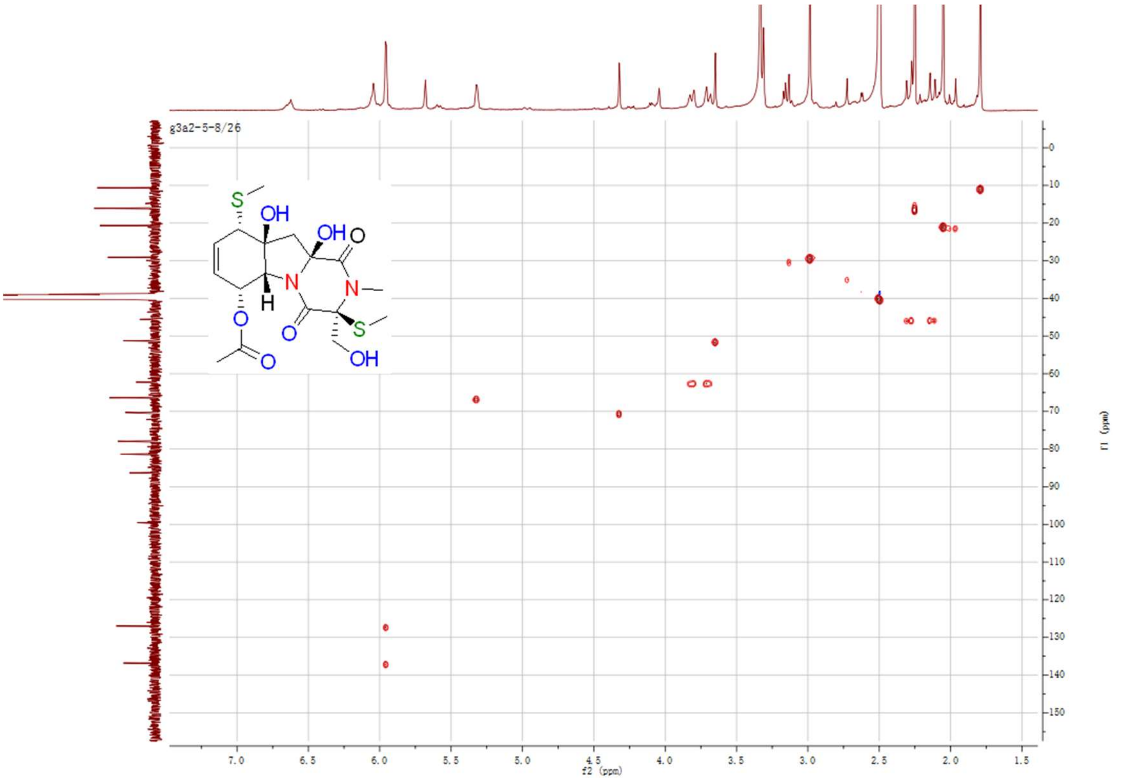

Figure S11. ^{1}H–^{1}H COSY spectrum of geospallin B (**2**) in DMSO-d_6.

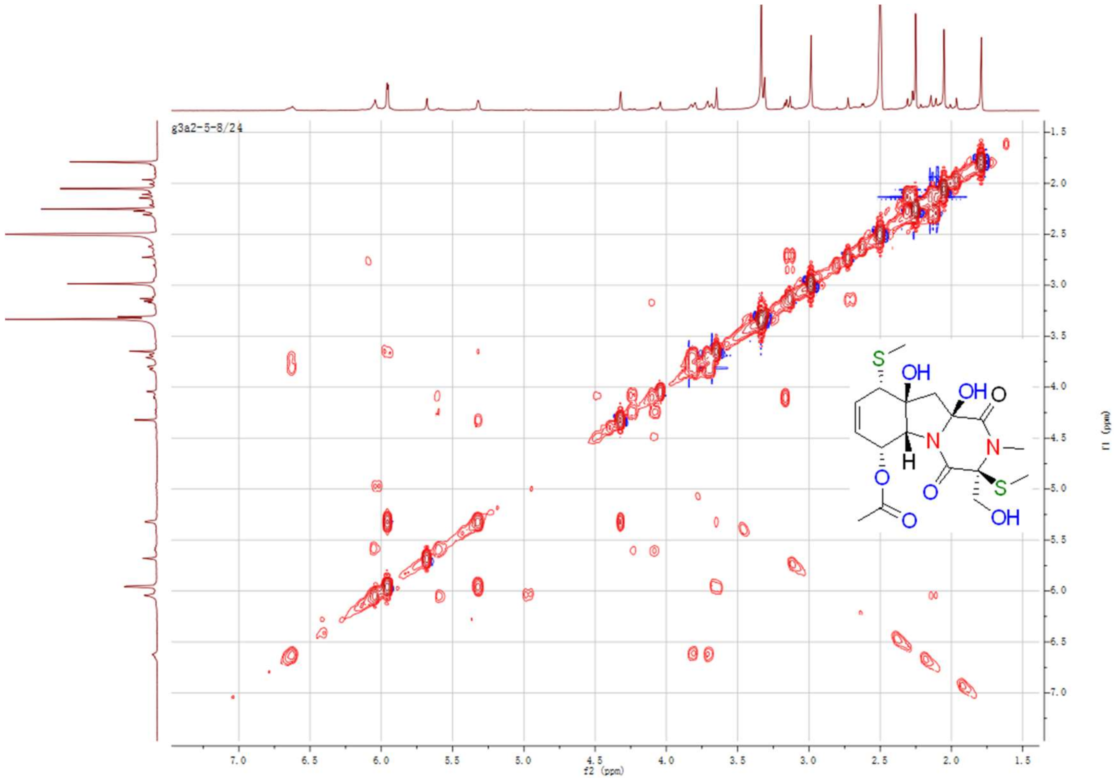

Figure S12. HMBC spectrum of geospallin B (**2**) in DMSO-d_6.

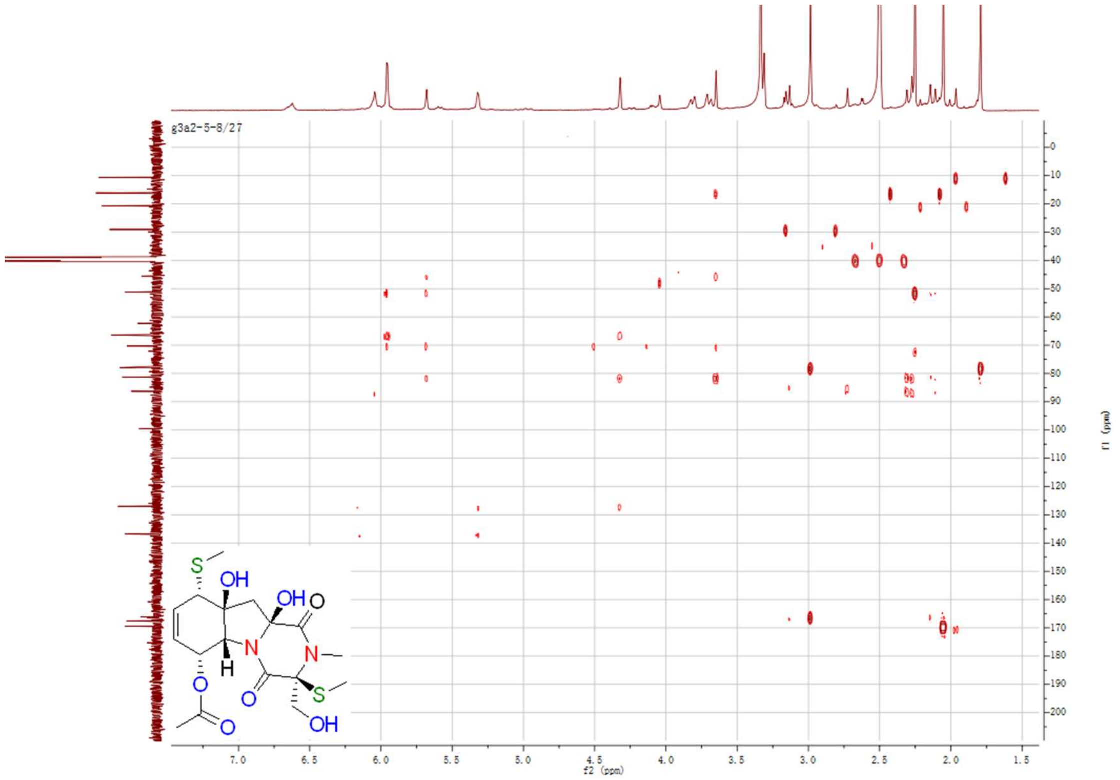

Figure S13. NOESY spectrum of geospallin B (**2**) in DMSO-d_6.

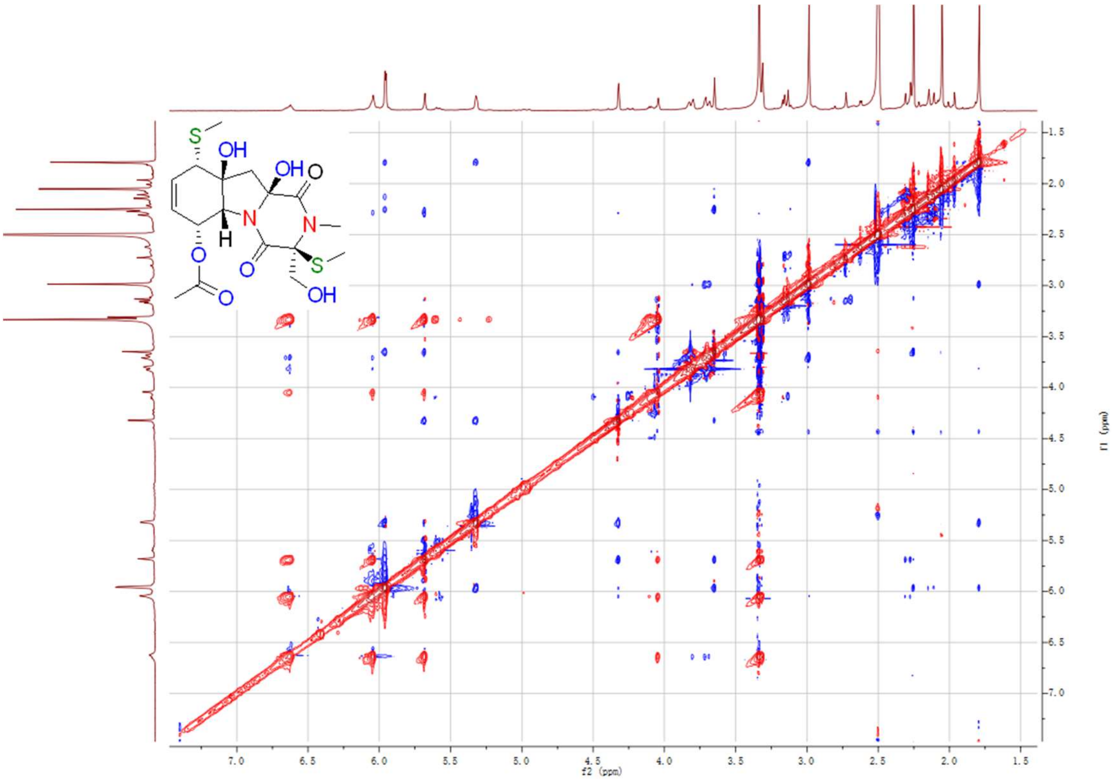

Figure S14. HRESIMS spectrum of geospallin B (**2**).

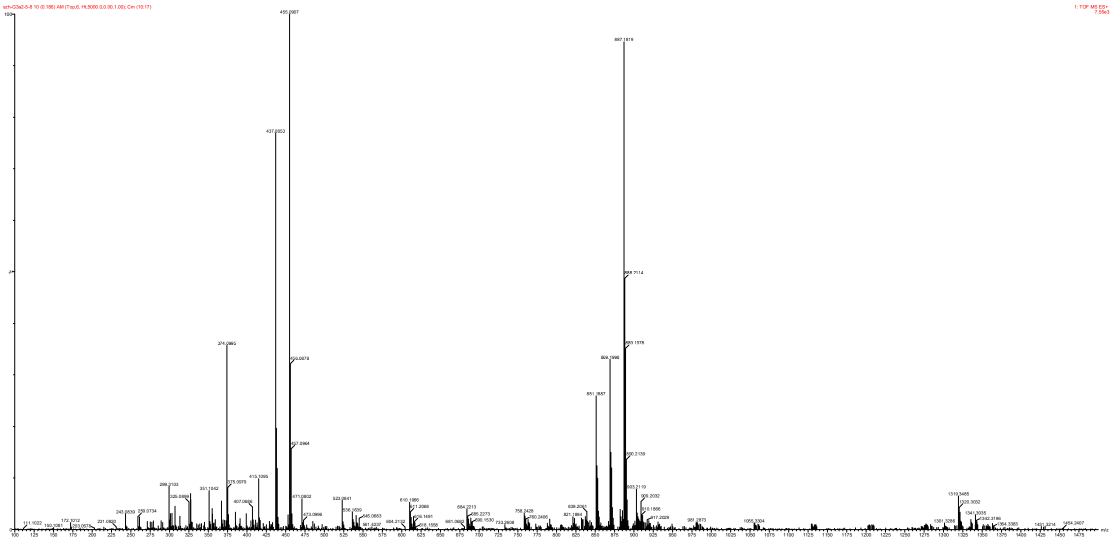

Figure S15. ^{1}H NMR spectrum of geospallin C (**3**) in DMSO-d_6.

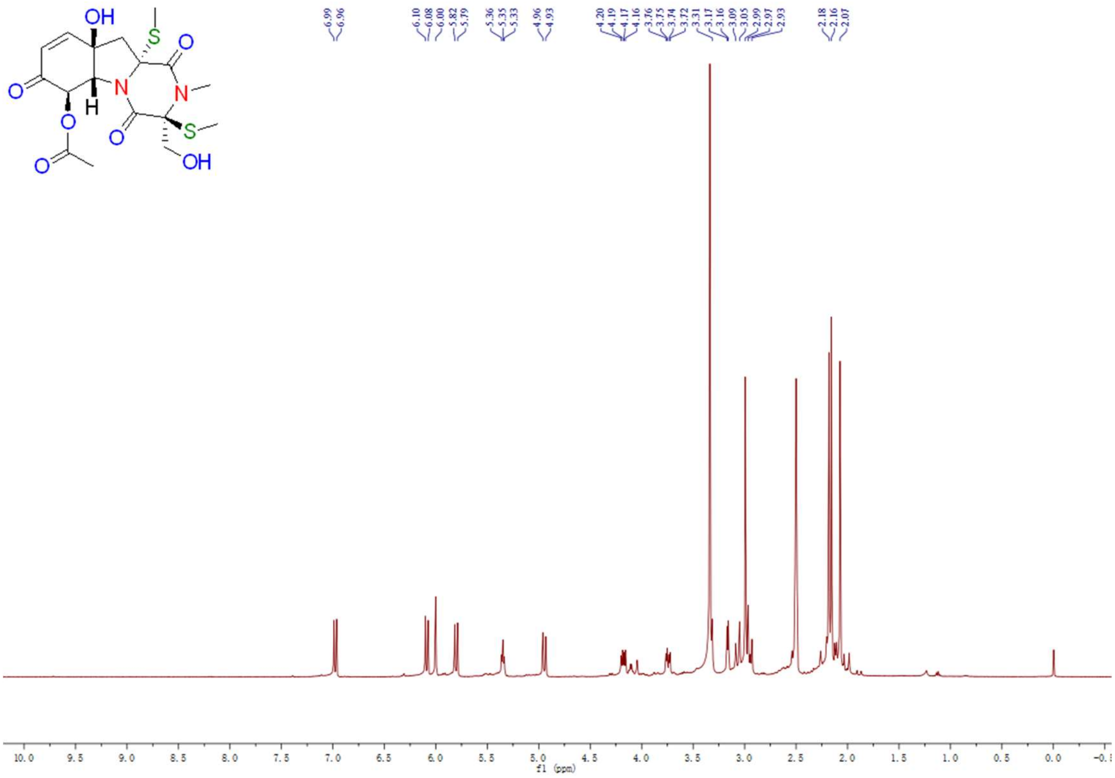

Figure S16. ^{13}C NMR spectrum of geospallin C (**3**) in DMSO-d_6.

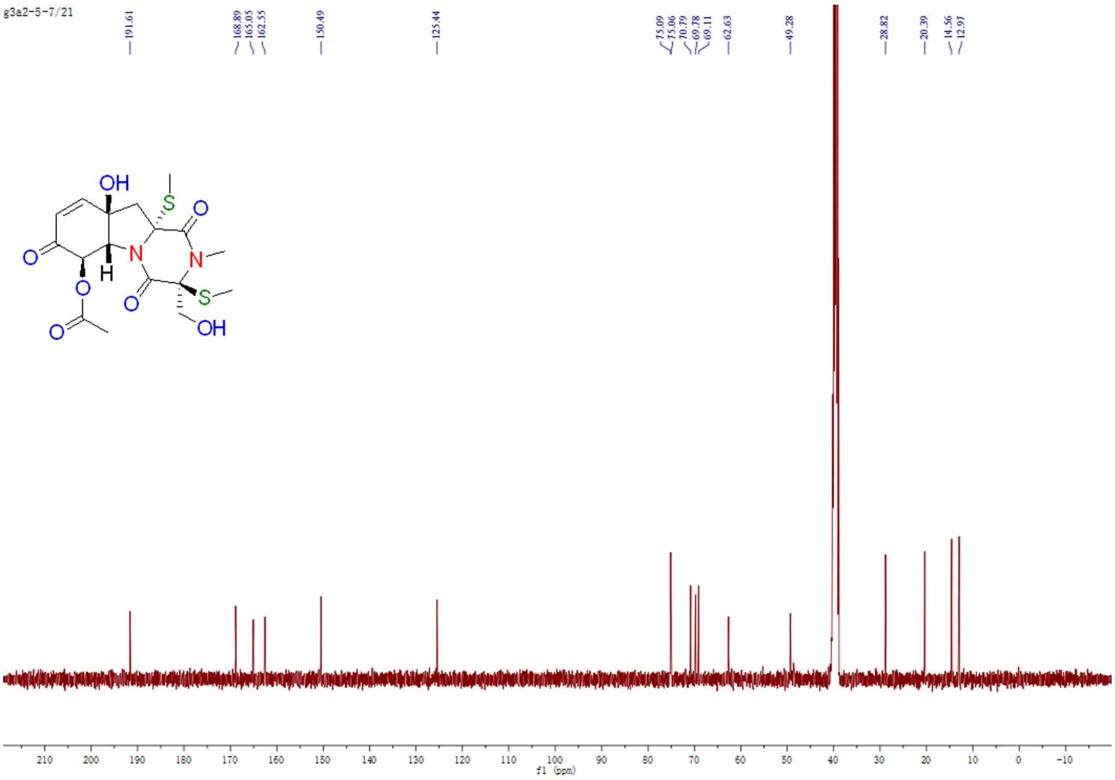

Figure S17. HSQC spectrum of geospallin C (**3**) in DMSO-d_6.

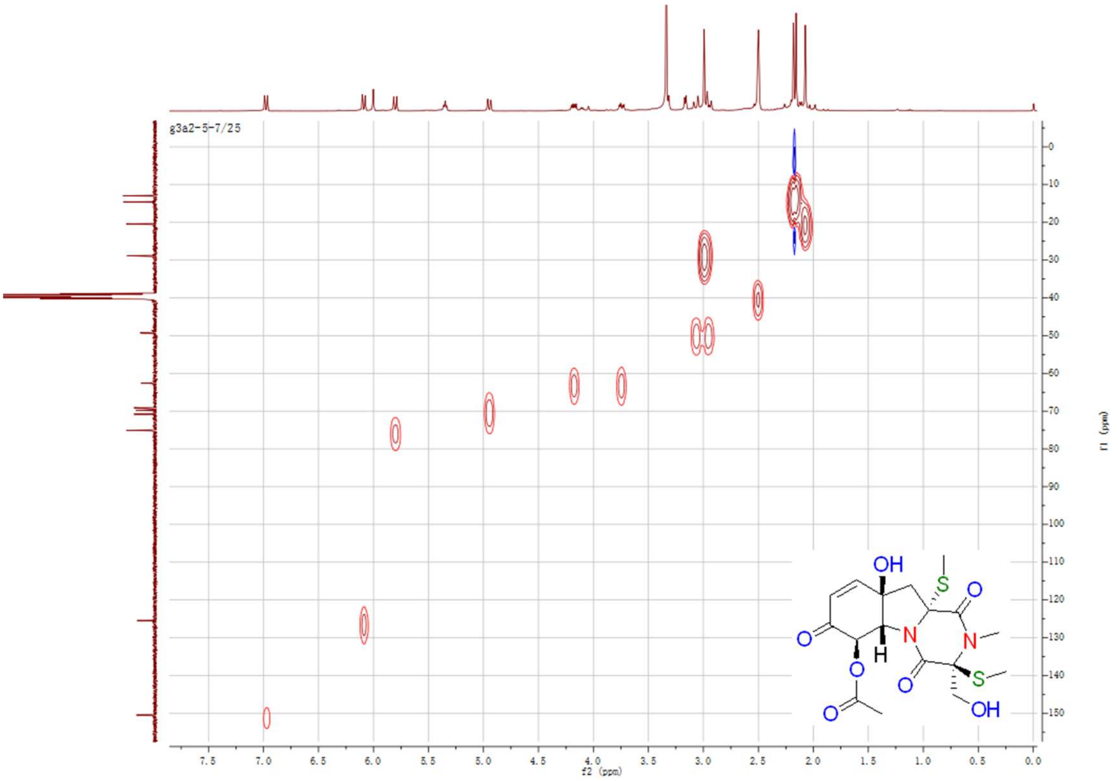

Figure S18. ^{1}H–^{1}H COSY spectrum of geospallin C (**3**) in DMSO-d_6.

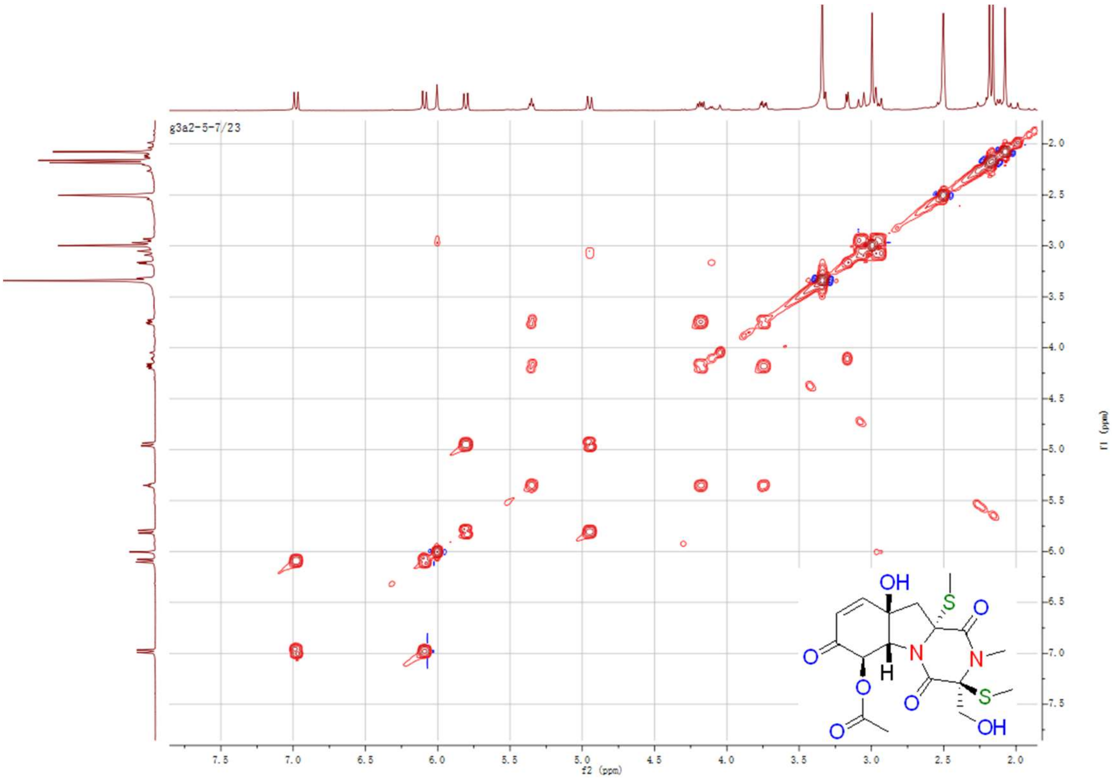

Figure S19. HMBC spectrum of geospallin C (**3**) in DMSO-d_6.

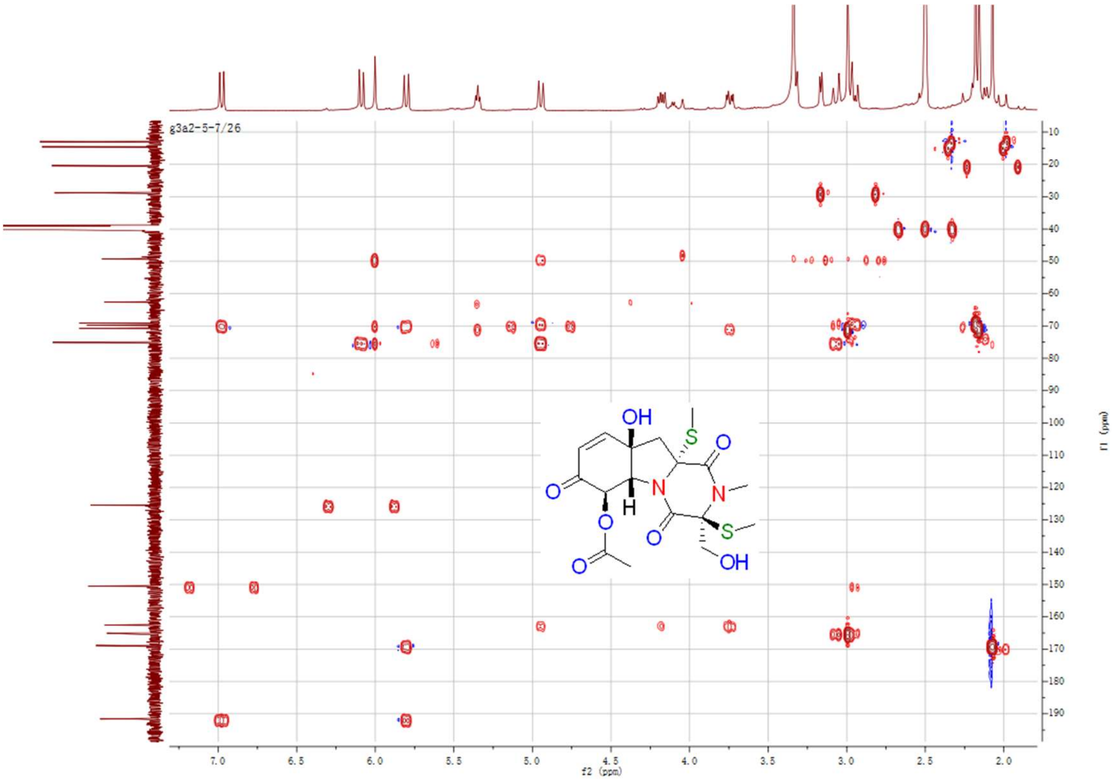

Figure S20. NOESY spectrum of geospallin C (**3**) in DMSO-d_6.

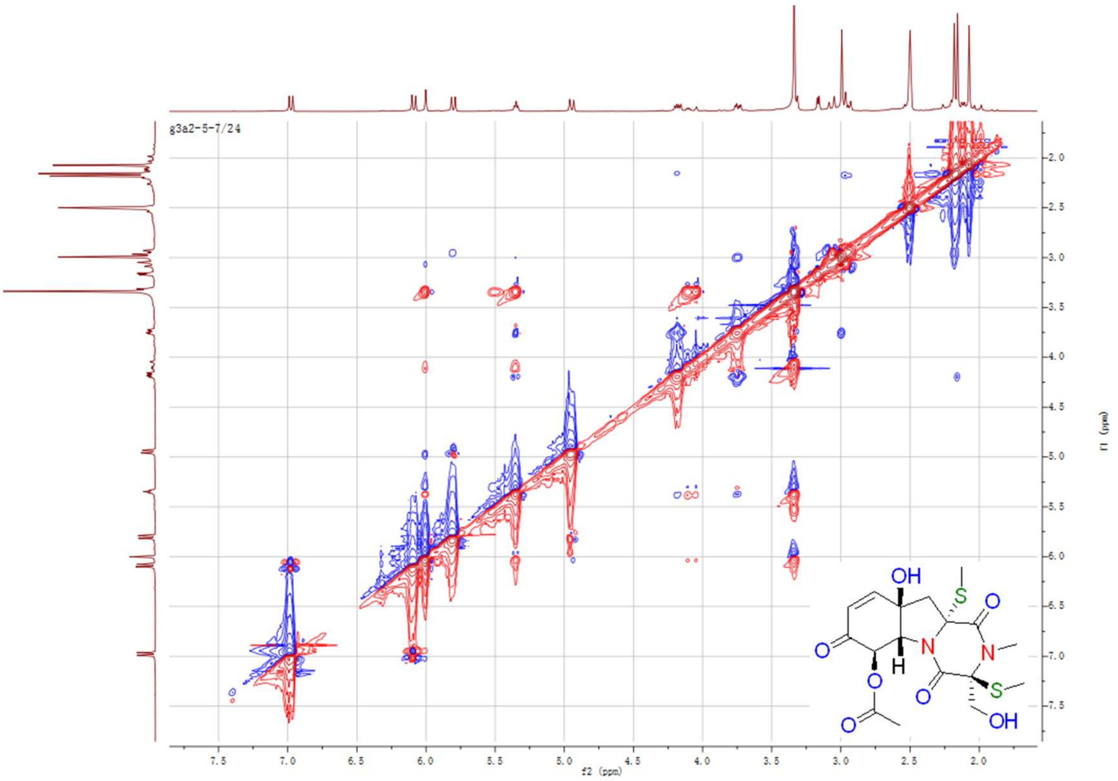

Figure S21. HRESIMS spectrum of geospallin C (**3**).

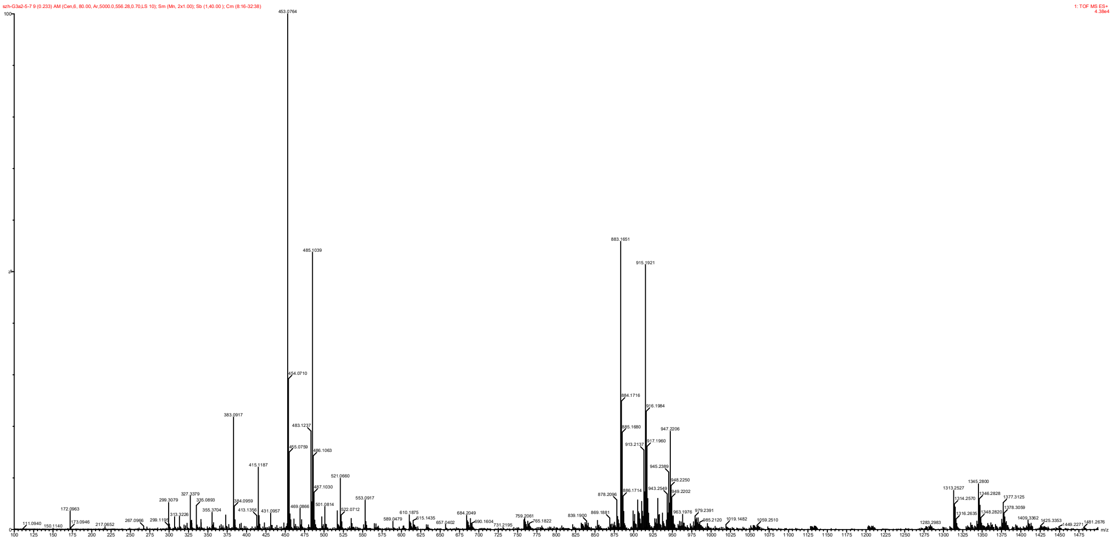

Figure S22. ^{1}H NMR spectrum of bisdethiobis (methylthio)gliotoxin (**4**) in CD_3OD.

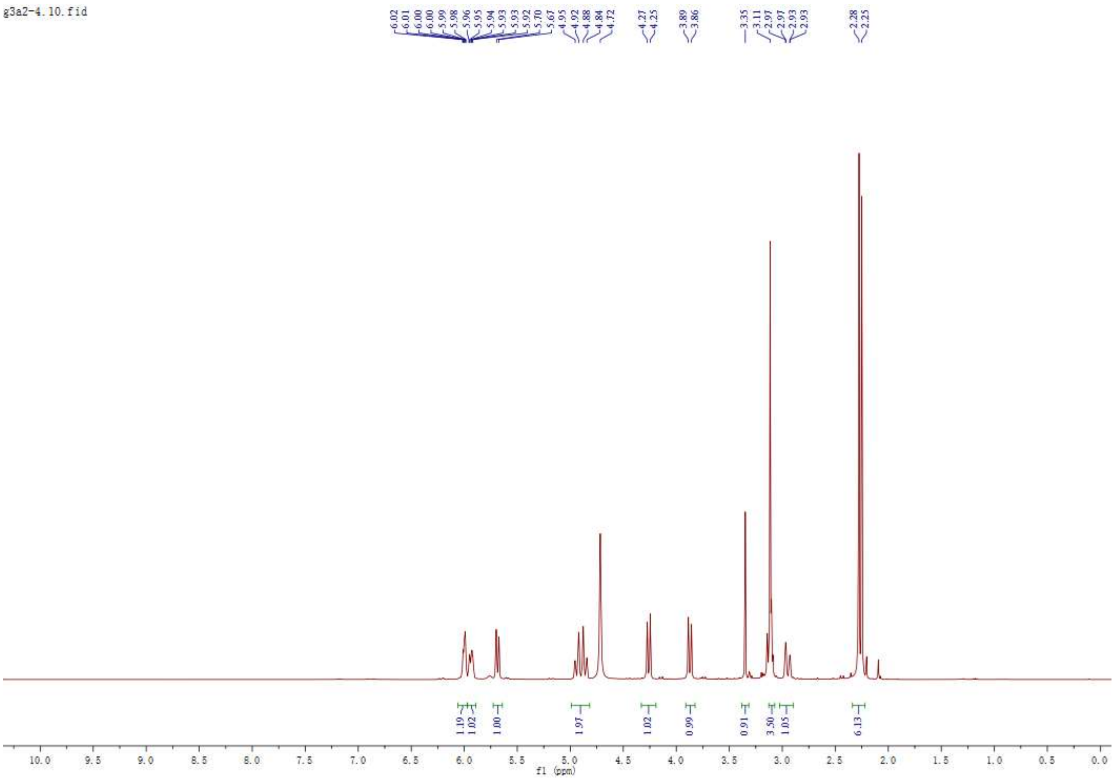

Figure S23. ^{13}C NMR spectrum of bisdethiobis (methylthio)gliotoxin (**4**) in CD_3OD.

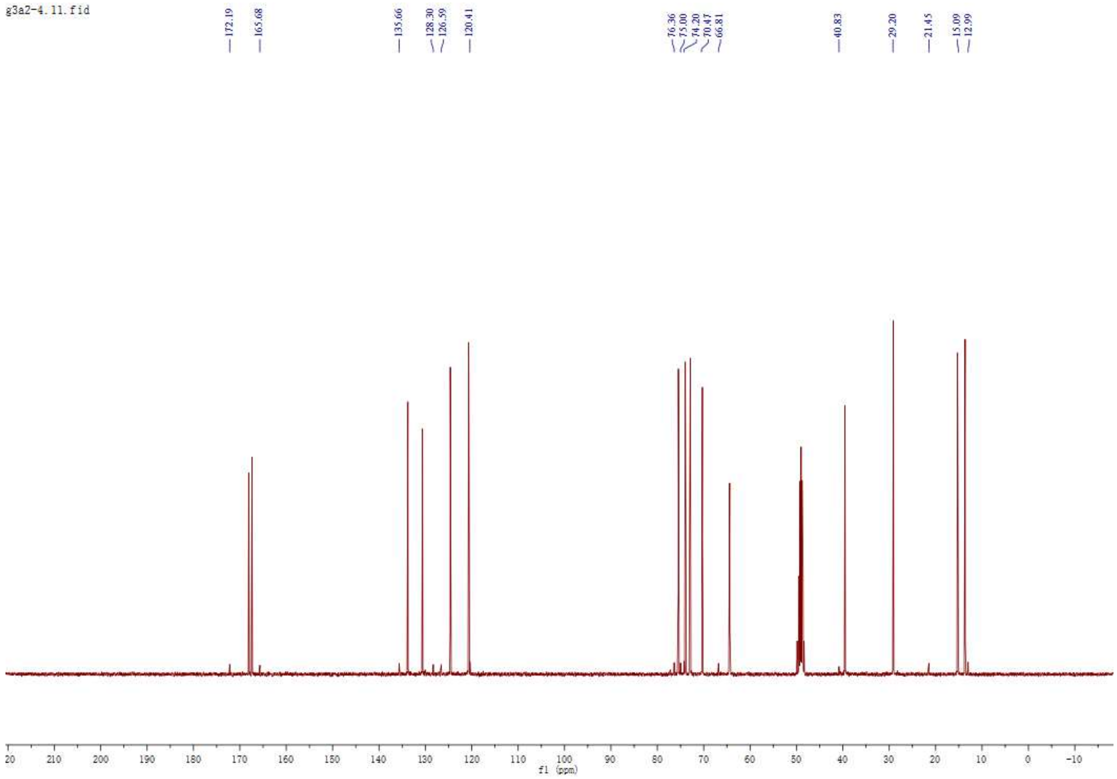

Figure S24. ^{1}H NMR spectrum of 6-acetylbis(methylthio)gliotoxin (**5**) in CD_3OD.

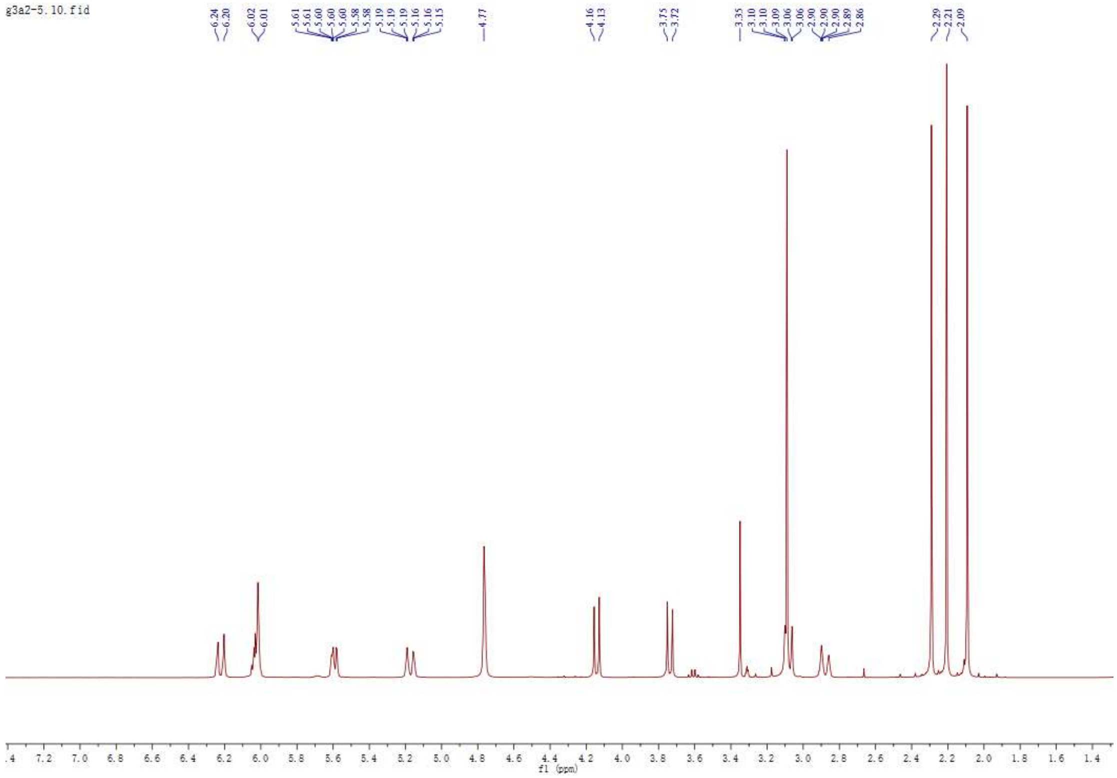

Figure S25. ^{13}C NMR spectrum of 6-acetylbis(methylthio)gliotoxin (**5**) in CD_3OD.

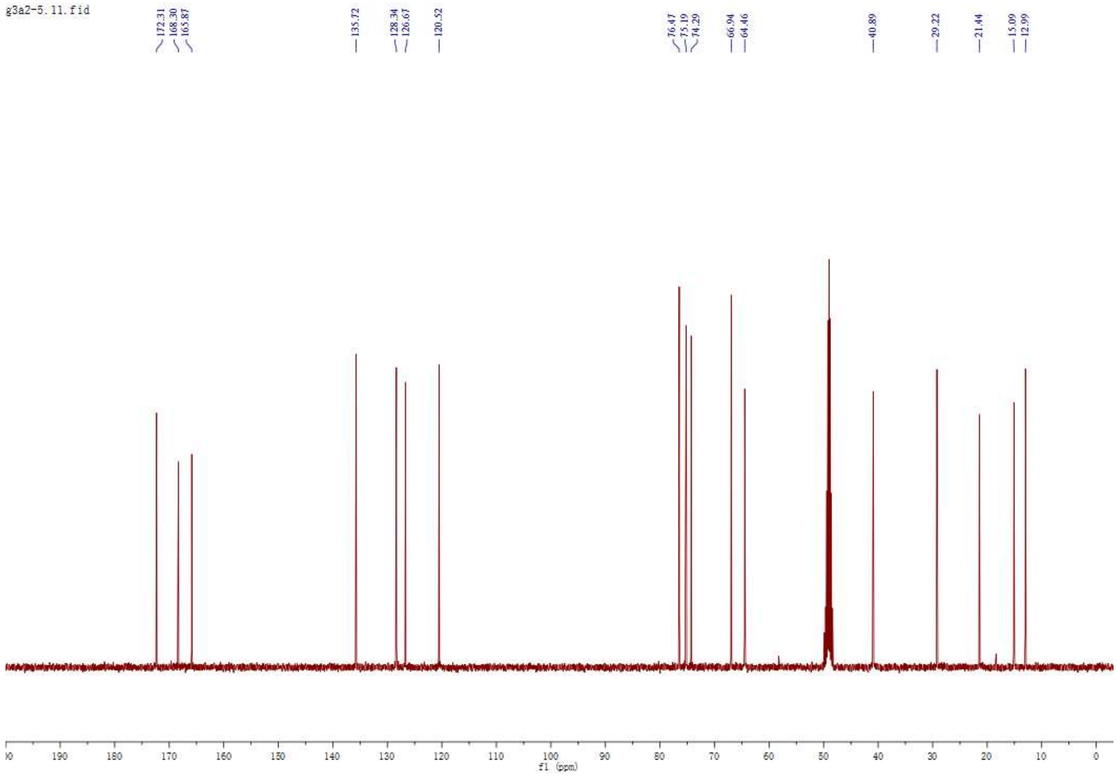

Figure S26. ^{1}H NMR spectrum of 6-deoxy-5a,6-didehydrogliotoxin (**6**) in DMSO-d_6.

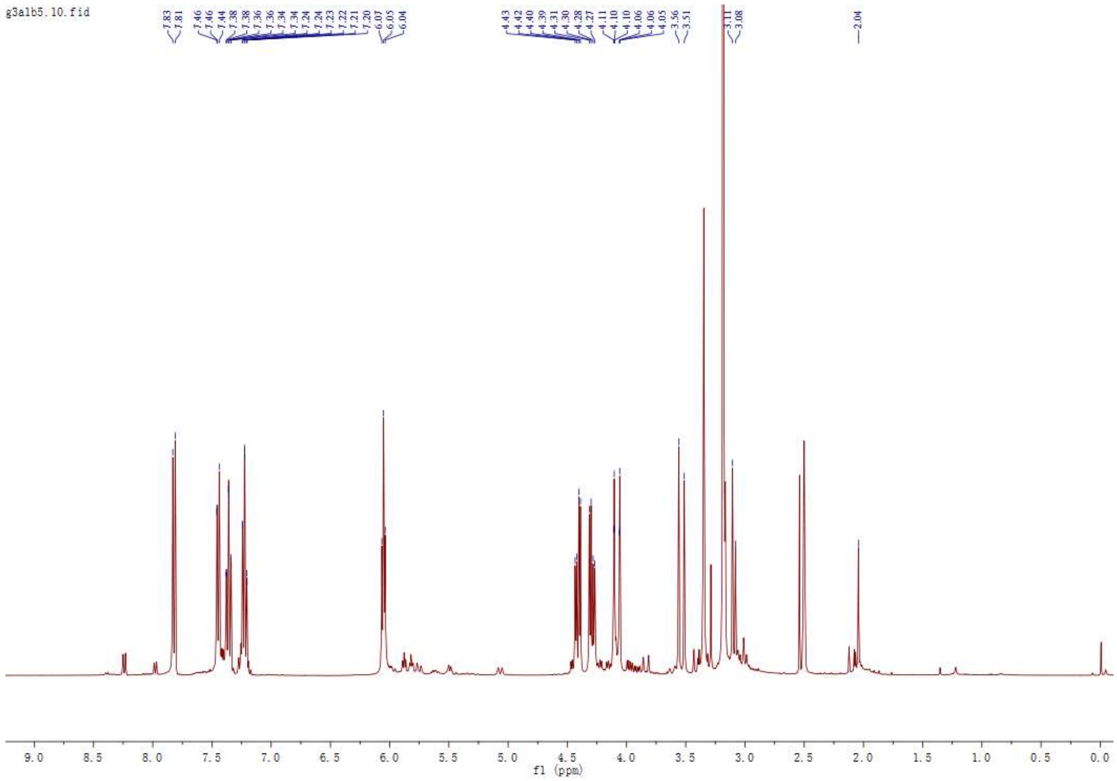

Figure S27. ^{13}C NMR spectrum of 6-deoxy-5a,6-didehydrogliotoxin (**6**) in DMSO-d_6.

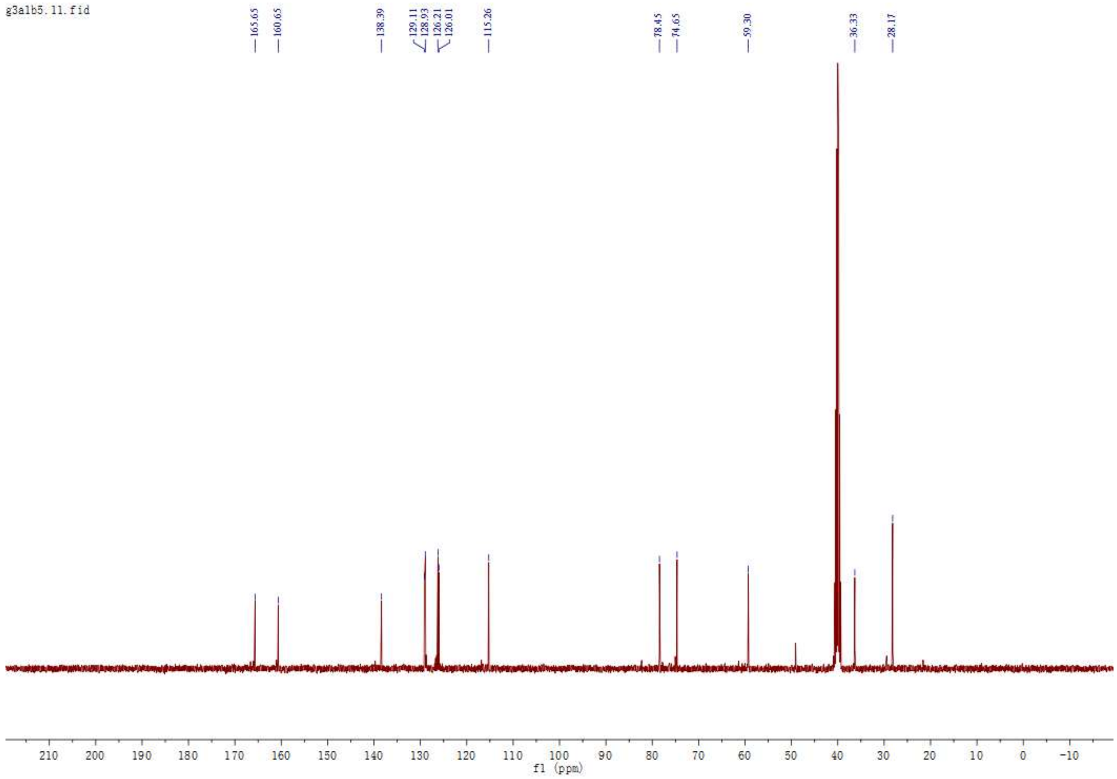

Figure S28. ^{1}H NMR spectrum of 5a,6-didehydrogliotoxin (**7**) in DMSO-d_6.

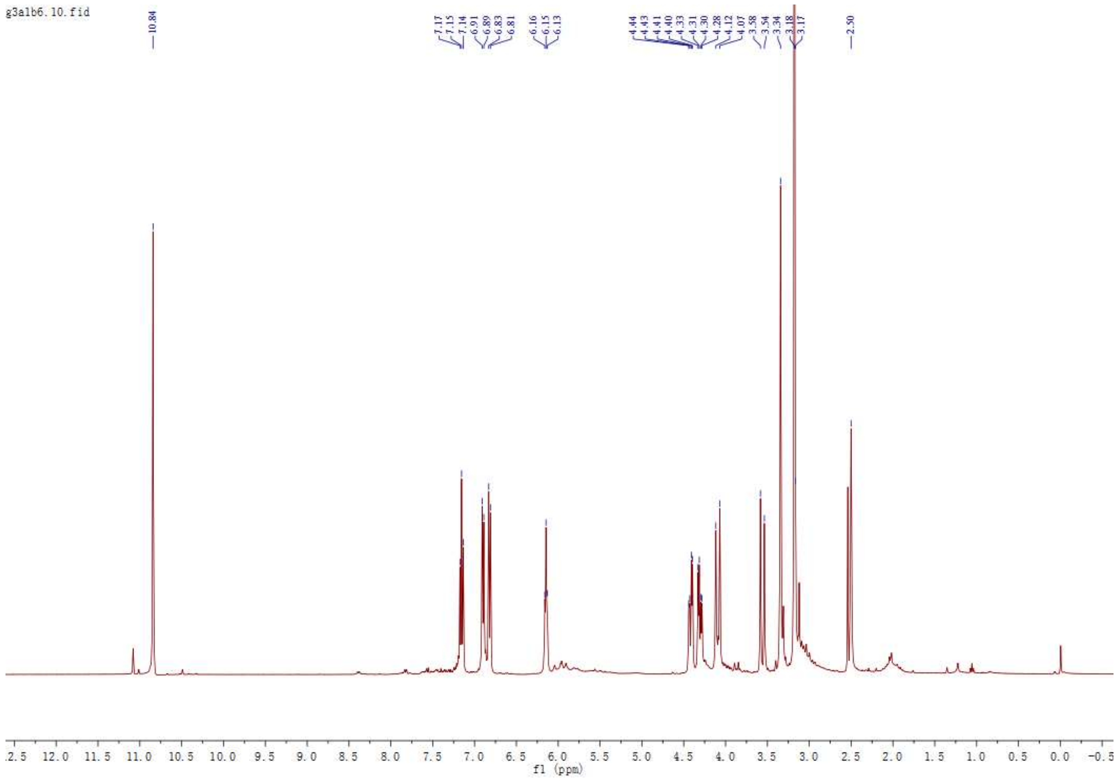

Figure S29. ^{13}C NMR spectrum of 5a,6-didehydrogliotoxin (**7**) in DMSO-d_6.

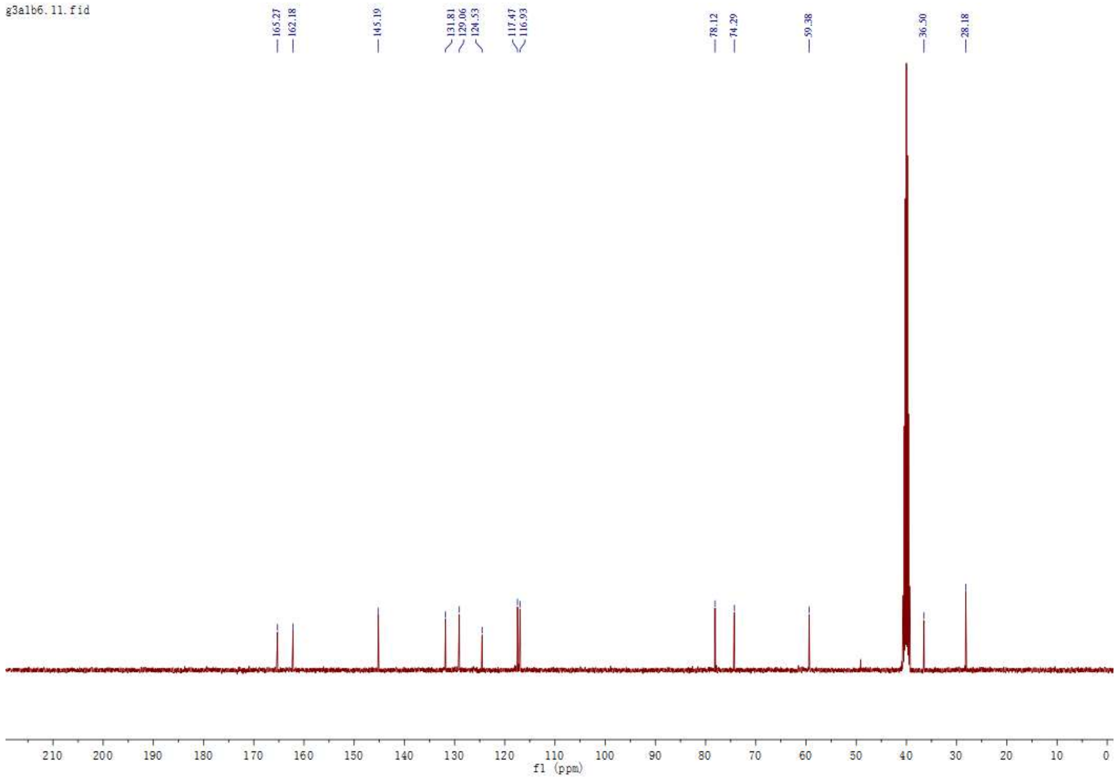

Figure S30. ^{1}H NMR spectrum of 6-(phenylmethyl)-(3R,6R)-2,5-piperazinedione (**8**) in CD_3OD.

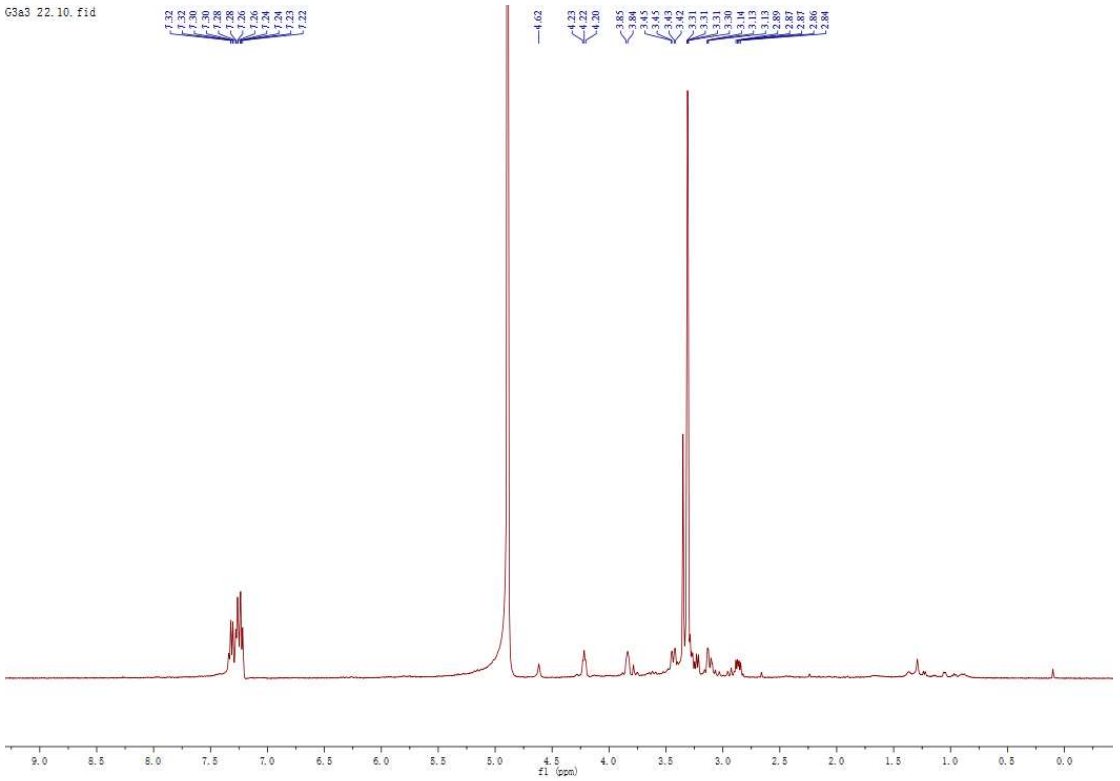

Figure S31. ^{13}C NMR spectrum of 6-(phenylmethyl)-(3R,6R)-2,5-piperazinedione (**8**) in CD_3OD.

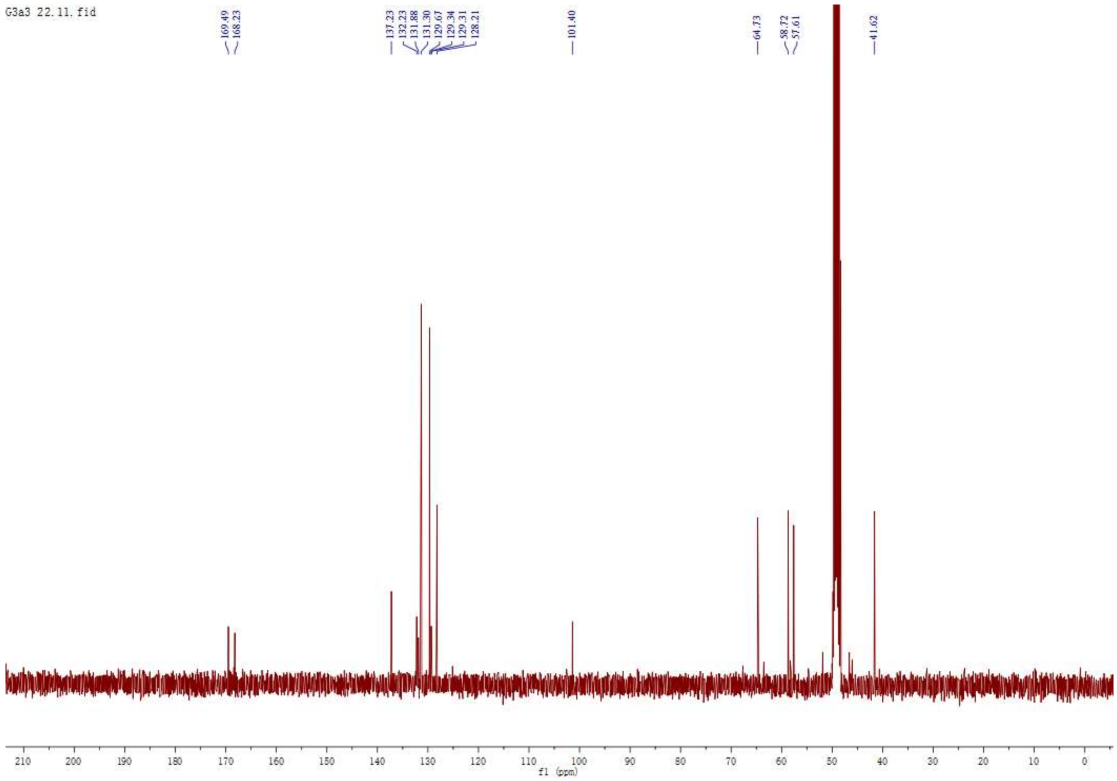

Figure S32. ^{1}H NMR spectrum of 3-(hydroxymethyl)-3,6-bis(methylthio)-6-(phenylmethyl)-(3R,6R)-2,5-piperazinedione (**9**) in DMSO-d_6.

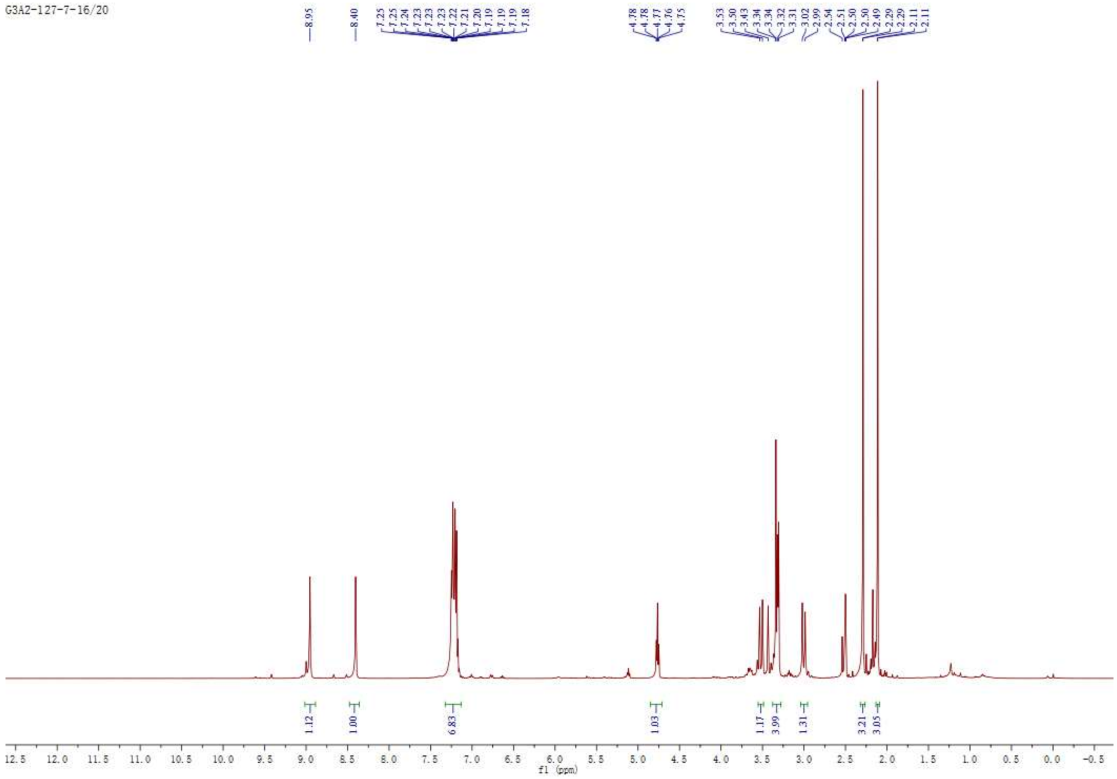

Figure S33. ^{13}C NMR spectrum of 3-(hydroxymethyl)-3,6-bis(methylthio)-6-(phenylmethyl)-(3R,6R)-2,5-piperazinedione (**9**) in DMSO-d_6.

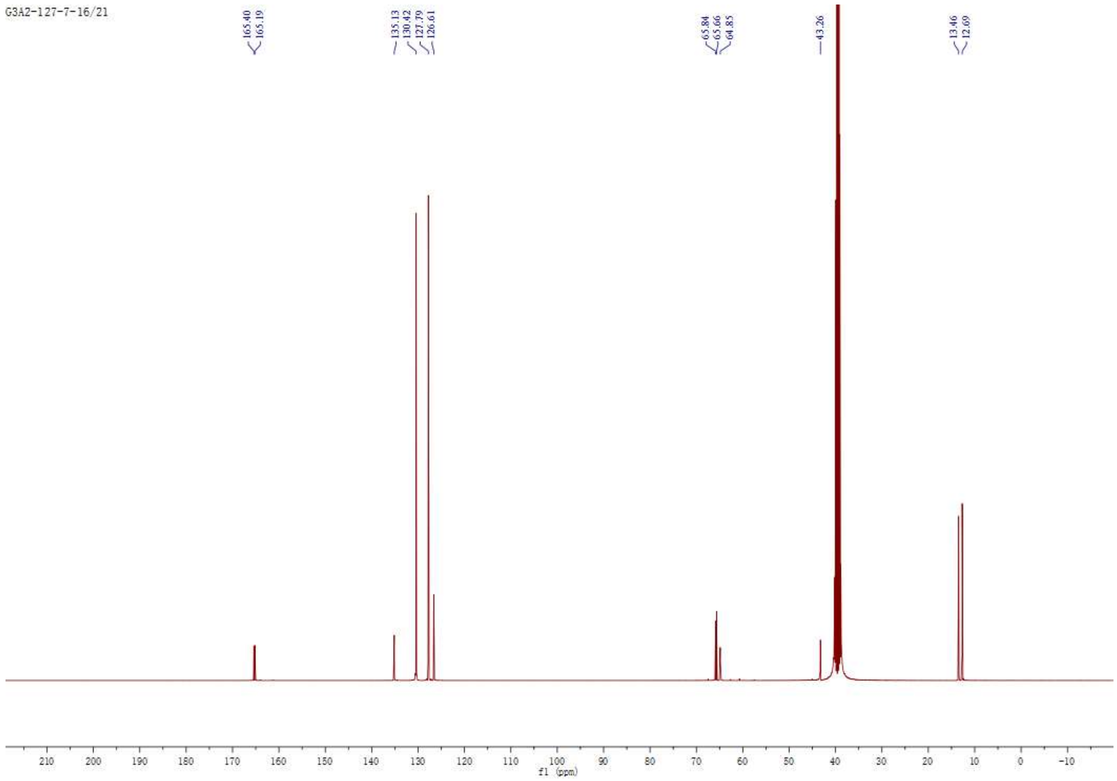

Figure S34. ^{1}H NMR spectrum of 3-(hydroxymethyl)-6-(methoxyl)-6-(phenylmethyl) -(3R,6R)-2,5-piperazinedione (**10**) in CD_3OD.

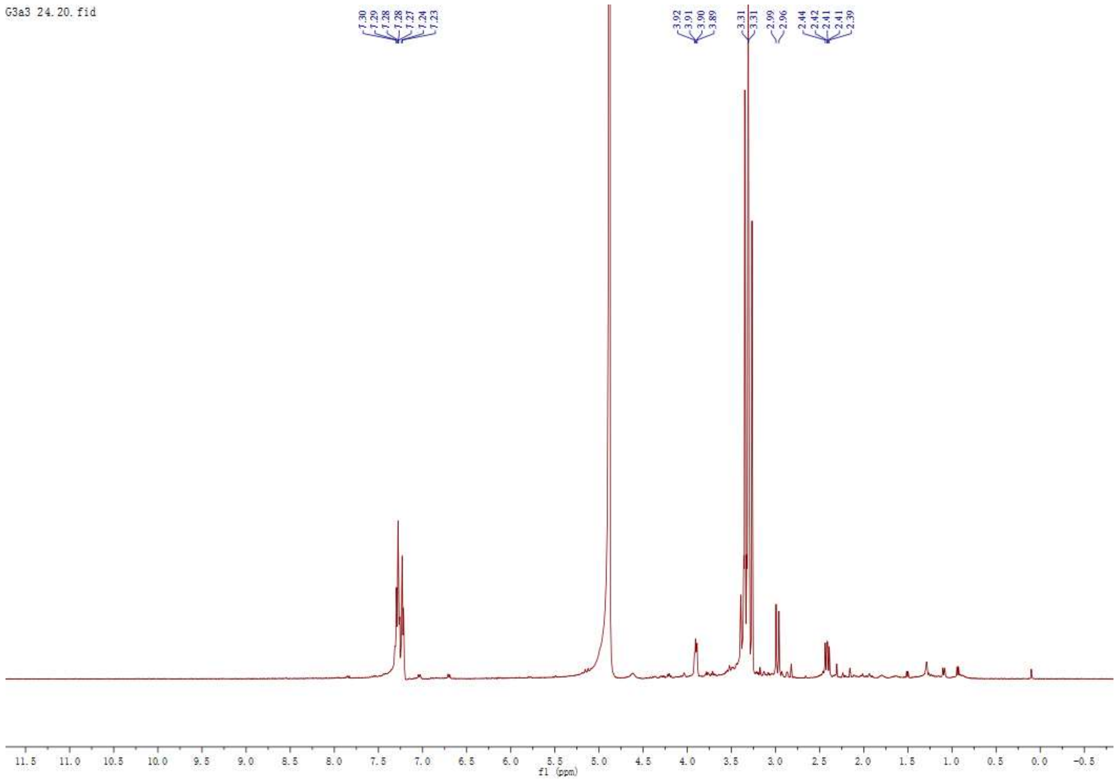

Figure S35. ^{13}C NMR spectrum of 3-(hydroxymethyl)-6-(methoxyl)-6-(phenylmethyl)-(3R,6R)-2,5-piperazinedione (**10**) in CD_3OD.

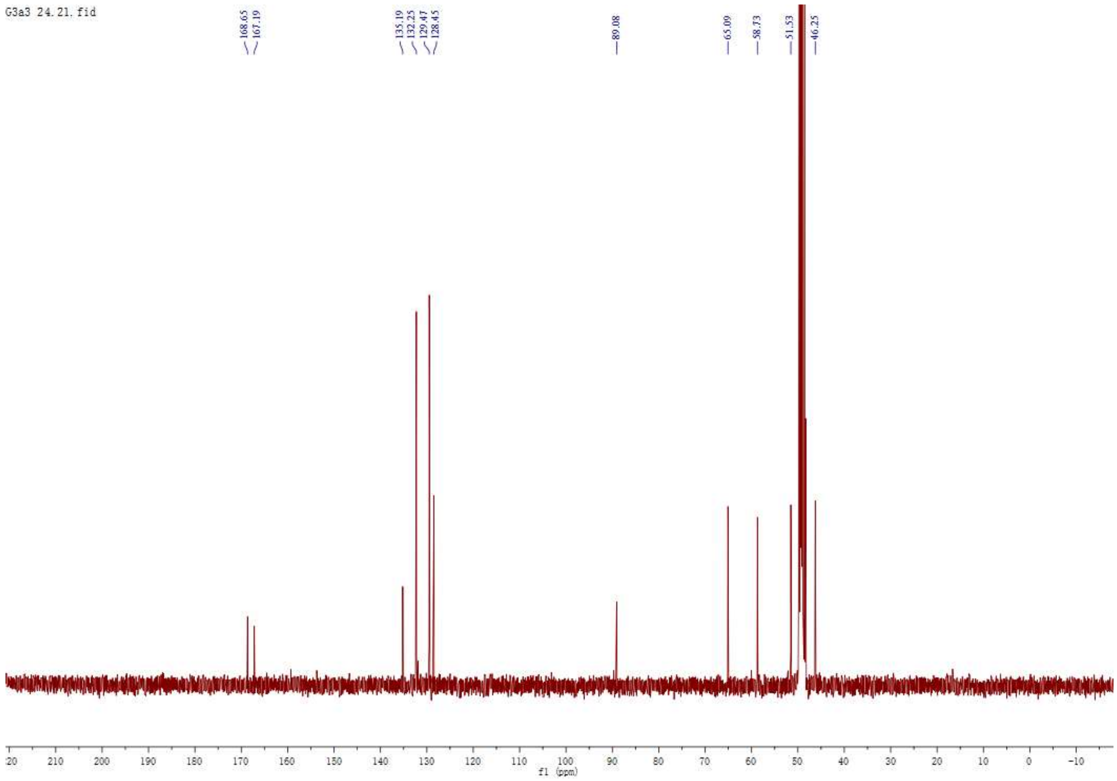

Figure S36. ^{1}H NMR spectrum of 5a,6-anhydrobisdethiobis(methylthio)gliotoxin (**11**) in CD_3OD.

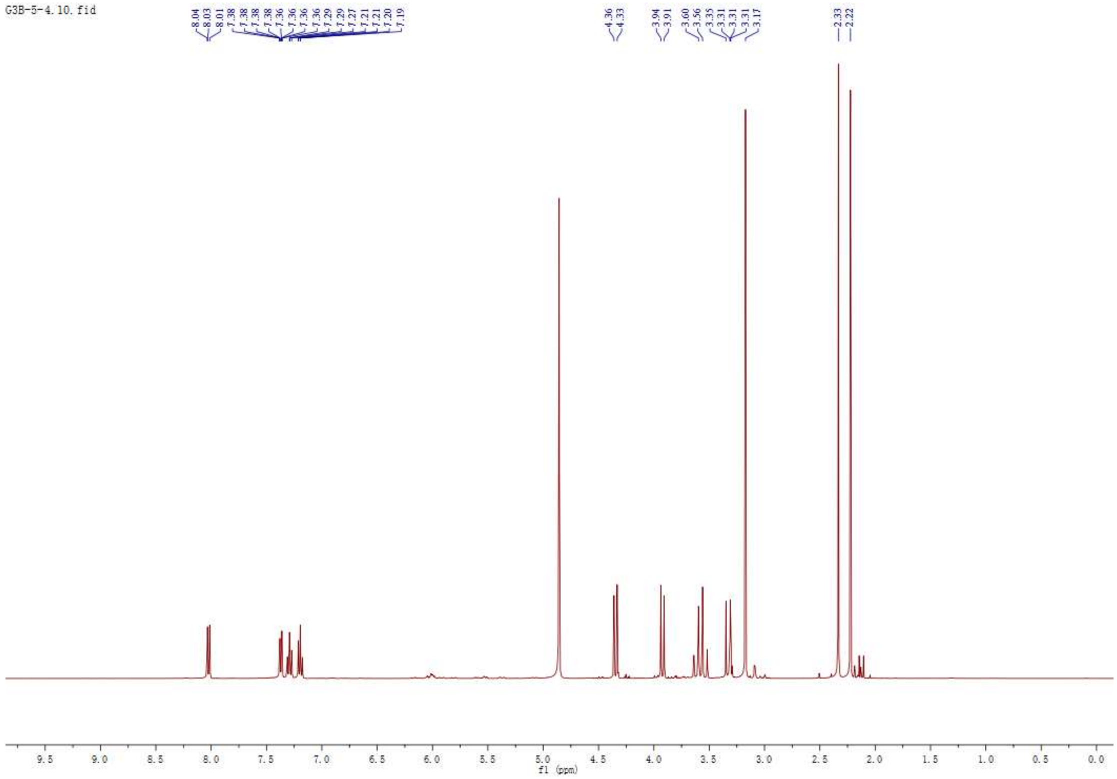

Figure S37. ^{13}C NMR spectrum of 5a,6-anhydrobisdethiobis(methylthio)gliotoxin (**11**) in CD_3OD.

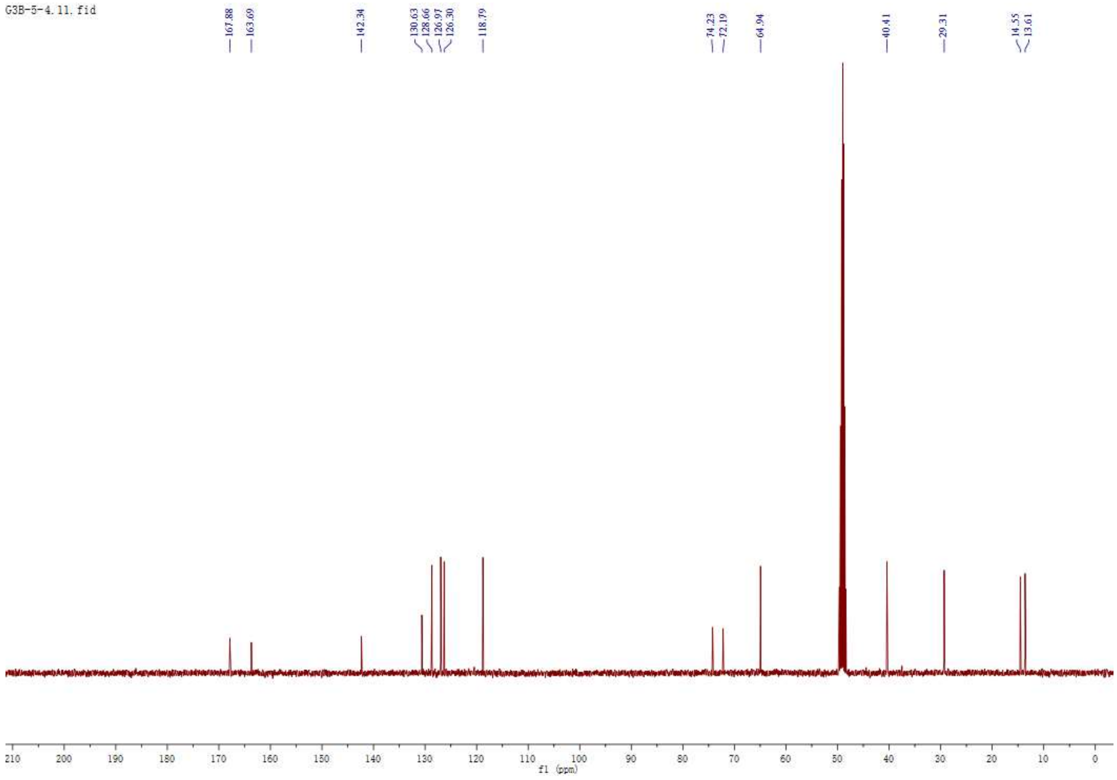

Figure S38. ^{1}H NMR spectrum of bisdethiobis (methylthio)gliotoxin (**12**) in CD_3OD.

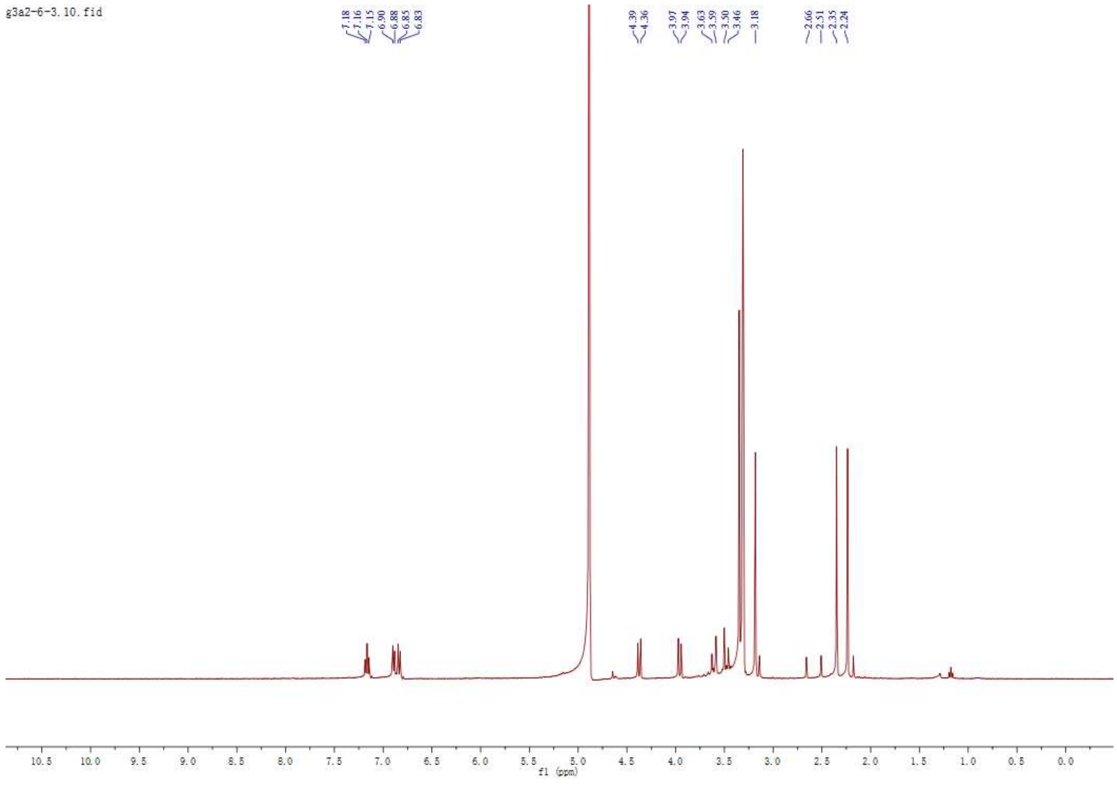

Figure S39. ^{13}C NMR spectrum of bisdethiobis (methylthio)gliotoxin (**12**) in CD_3OD.

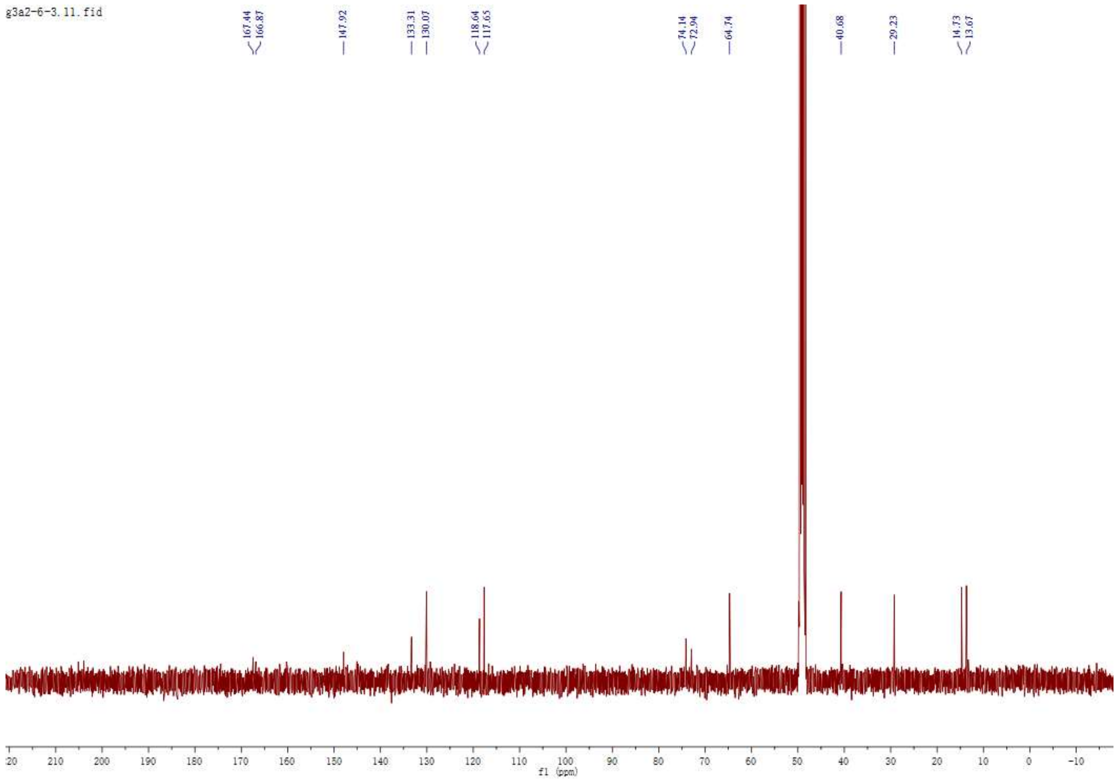

Figure S40. CD spectrum of geospallin A (**1**).

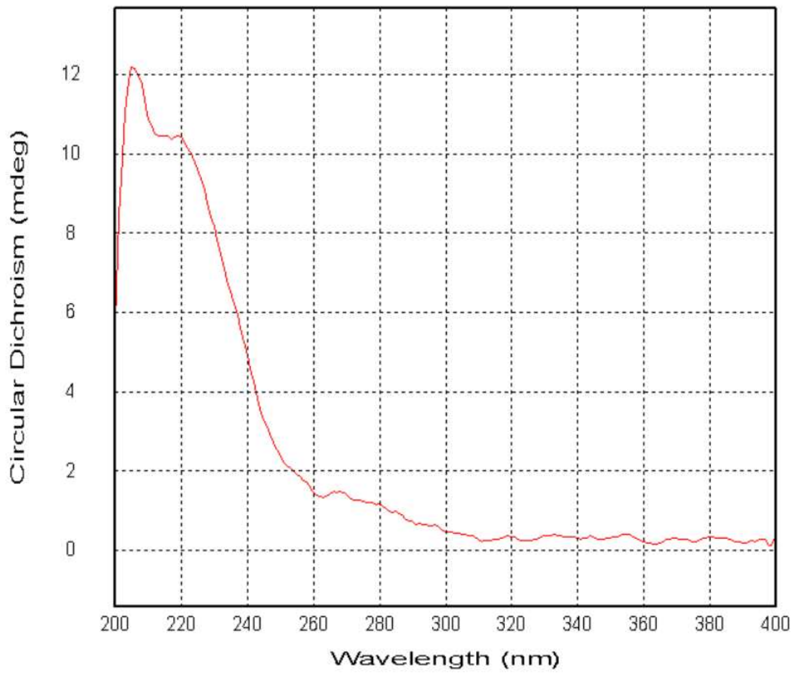

Figure S41. CD spectrum of geospallin B (**2**).

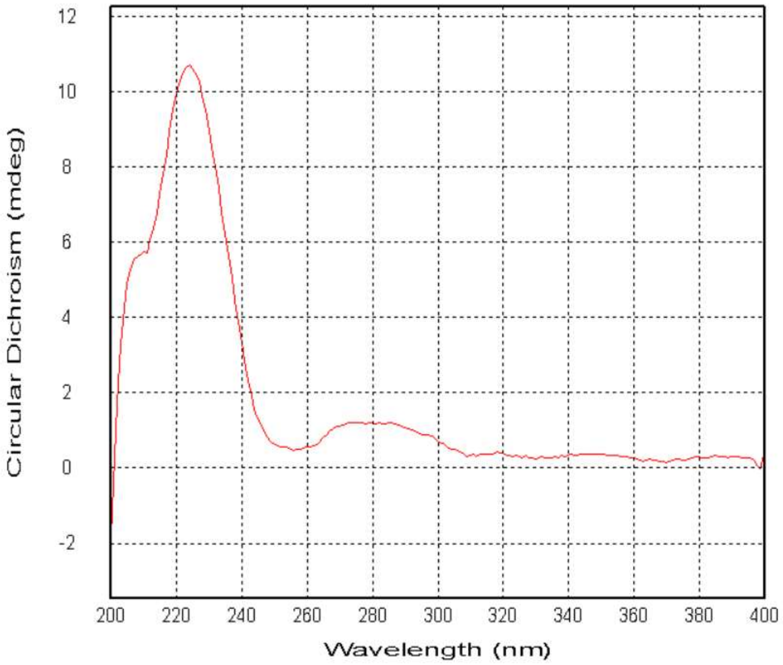

Figure S42. CD spectrum of geospallin C (**3**).

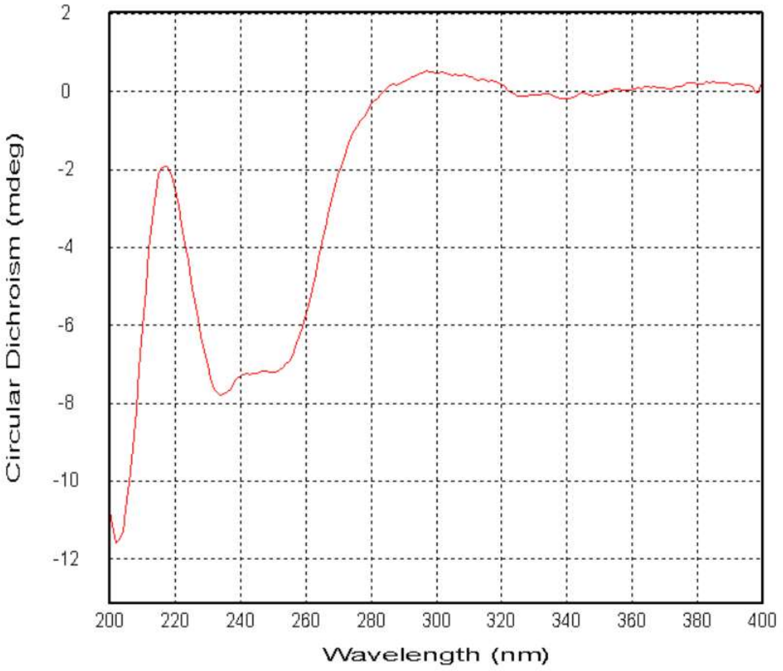

Figure S43. ESI$^{(+)}$MS spectrum of geospallin A (**1**).

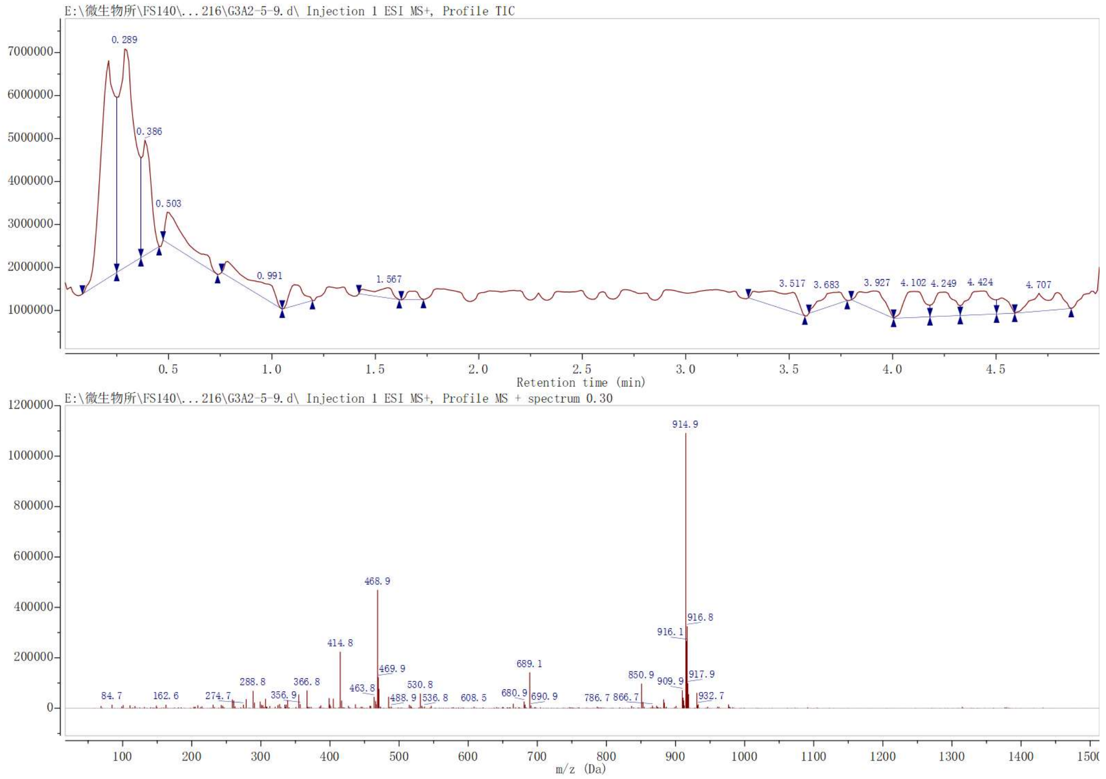

Figure S44. ESI$^{(–)}$MS spectrum of geospallin B (**2**).

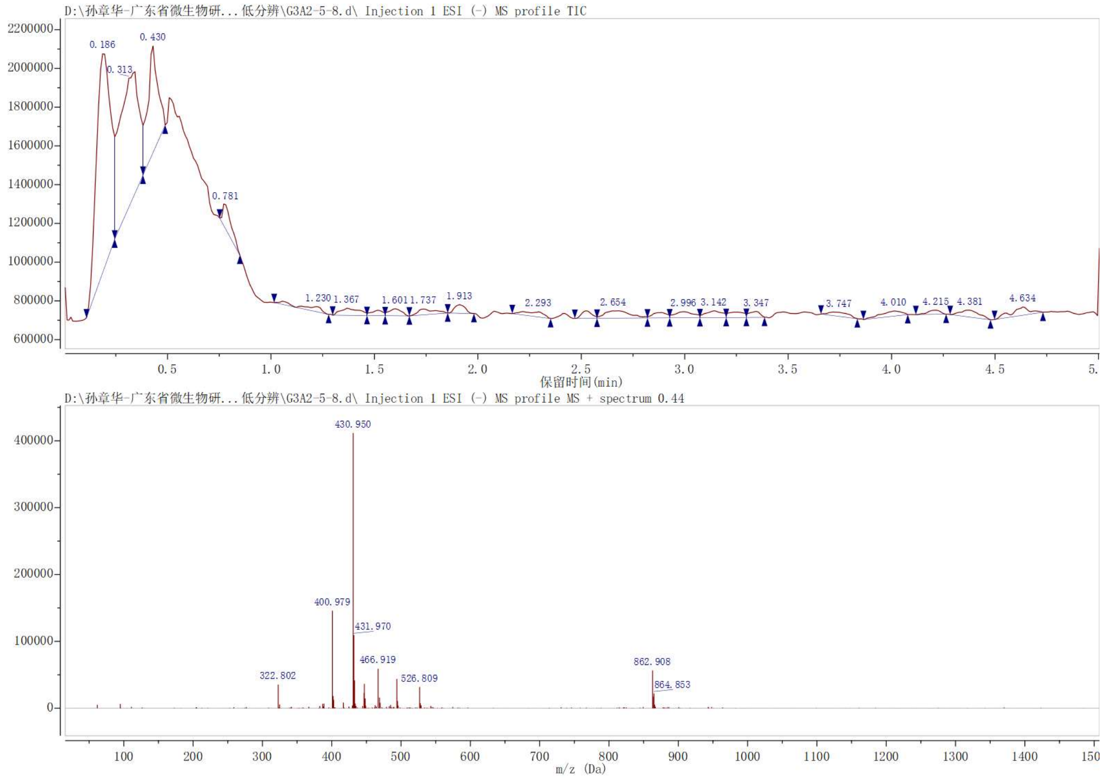

Figure S45. ESI$^{(–)}$MS spectrum of geospallin C (**3**).

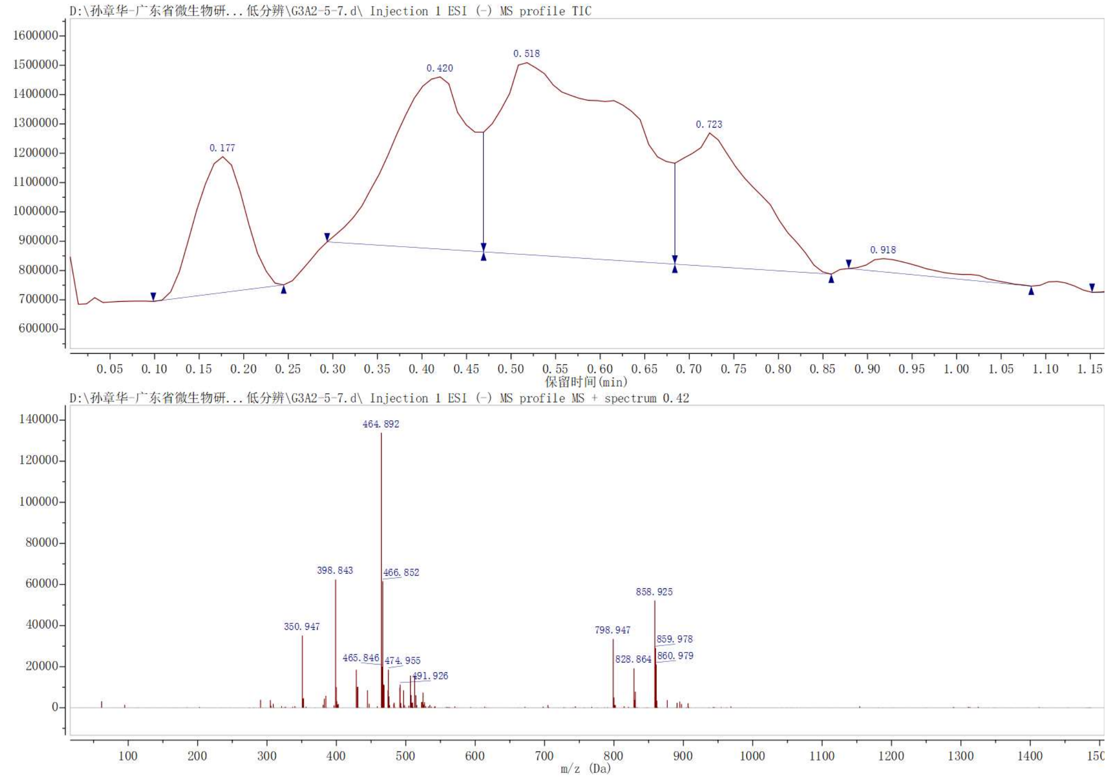

Figure S46. Experimental ECD spectra of geospallin B (**2**) in MeOH and calculated ECD spectra of (3*R*, 6*R*, 7*R*, 10*S*, 11*R*, 13*R*)-**2**, (3*R*, 6*R*, 7*R*, 10*S*, 11*R*, 13*S*)-**2**, (3*S*, 6*S*, 7*S*, 10*R*, 11*S*, 13*R*)-**2**, and (3*S*, 6*S*, 7*S*, 10*R*, 11*S*, 13*S*)-**2**.

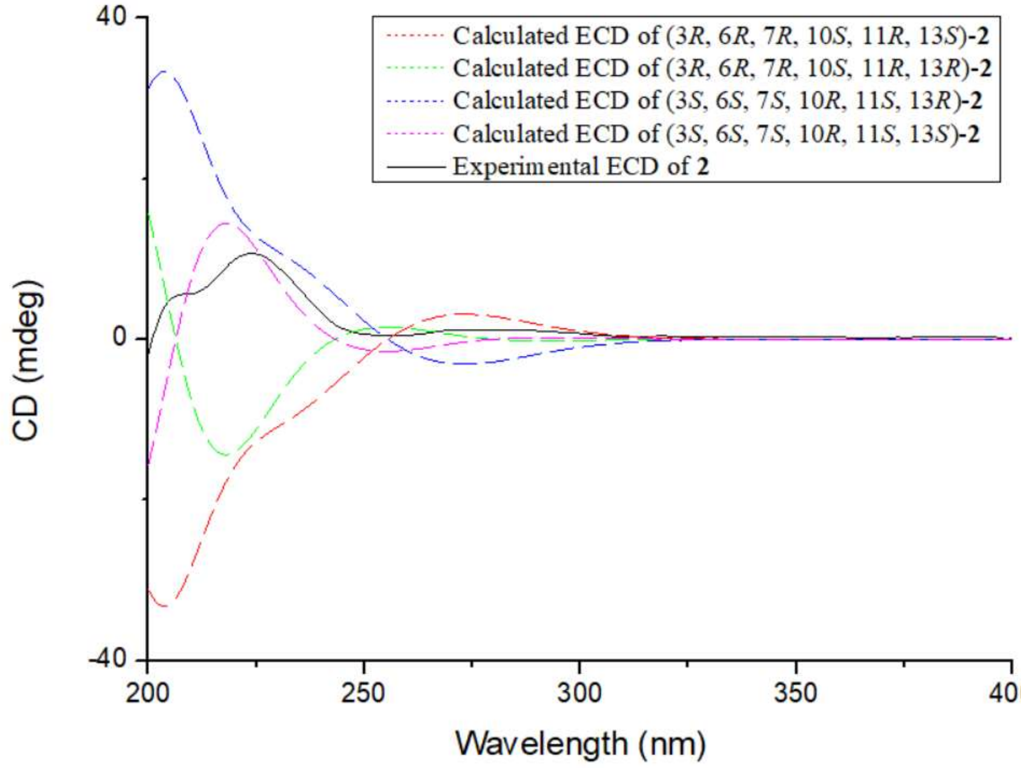

S47. ECD spectrum calculation.

The 3D structures were generated by Discover Studio 2.5. The conformational search was performed by the Conformer Searching module[1] of Open Babel 2.4.1[2] using a genetic algorithm with the MMFF94 molecular mechanics force field. Overall, 30 conformations for each configuration were obtained and then optimized by using DFT at the b3lyp/6-311+g(2d,p) level. The b3lyp/6-311+g(2d,p) harmonic vibrational frequencies were also calculated to confirm their stability. Due to the rigid skeleton, these optimized conformers had no significant differences. The most stable conformer, which had no imaginary frequency, was subsequently submitted to ECD calculations by TDDFT at the b3lyp/6-311+g(2d,p) level. The solvent effects were taken into account by the integral equation formalism polarizable continuum model (IEFPCM, methanol). All calculations were performed with the Gaussian 16 A.03 program[3]. The calculated ECD spectrum were drawn by SpecDis 1.7.1[4] software with a UV shift to the ECD spectrum.

1. O'Boyle, N. M.; Vandermeersch, T.; Flynn, C. J.; Maguire, A. R.; Hutchison, G. R., Confab - Systematic generation of diverse low-energy conformers. *J. Cheminform.* **2011,** *3*, 8.
2. O'Boyle, N. M.; Banck, M.; James, C. A.; Morley, C.; Vandermeersch, T.; Hutchison, G. R., Open Babel: An open chemical toolbox. *J. Cheminform.* **2011,** *3*, 33.
3. Frisch, M. J.; Trucks, G. W.; Schlegel, H. B.; Scuseria, G. E.; Robb, M. A.; Cheeseman, J. R.; Scalmani, V.; Barone, H.; Petersson, G. A.; Nakatsuji, X.; Li, M.; Caricato, J.; Marenich, A. V.; Bloino, R.; Janesko, B. G.; Gomperts, B.; Mennucci, D.; Hratchian, H. P.; Ortiz, J. V.; Izmaylov, A. F.; Sonnenberg, J. L.; Williams-Young, F.; Ding, F.; Lipparini, F.; Egidi, J.; Goings, B.; Peng, A.; Petrone, T.; Henderson, D.; Ranasinghe, J.; Zakrzewski, V. G.; Gao, N.; Rega, G.; Zheng, W.; Liang, M.; Hada, M.; Ehara, K.; Toyota, R.; Fukuda, J.; Hasegawa, M.; Ishida, T.; Nakajima, Y.; Honda, O.; Kitao, H.; Nakai, T.; Vreven, K.; Throssell, F.; Montgomery, J. A.; Jr., P., J. E. ; Ogliaro, R.; Bearpark, M. J.; Heyd, J. J.; Brothers, E. N.; Kudin, K. N.; Staroverov, V. N.; Keith, T. A.; Kobayashi, J.; Normand, K.; Raghavachari, J.; Rendell, A. P.; Burant, J. C.; Iyengar, S. S.; Tomasi, M.; Cossi, M.; Millam, J. M.; Klene, C.; Adamo, R.; Cammi, K.; Ochterski, J. W.; Martin, R. L.; Morokuma, O.; Farkas; Foresman, J. B.; J., F. D., Gaussian 16, Revision A.03. *Gaussian, Inc., Wallingford CT* **2016**.
4. Bruhn, T.; Schaumloffel, A.; Hemberger, Y.; Bringmann, G., SpecDis: quantifying the comparison of calculated and experimental electronic circular dichroism spectrum. *Chirality* **2013,** *25* (4), 243-9.
